# Biochemical impact of ALAEm supplementation in late gestation on the reproductive performance of sows

**DOI:** 10.3389/fvets.2025.1548263

**Published:** 2025-04-23

**Authors:** Linlu Zhao, Jin Zhang, Jieyi He, Xingbin Ma, Zhichao Yu, Yanhong Yong, Youquan Li, Xianghong Ju, Xiaoxi Liu

**Affiliations:** Department of Veterinary Medicine, College of Coastal Agricultural Sciences, Guangdong Ocean University, Zhanjiang, China

**Keywords:** *Angelica sinensis* (Oliv.) Diels, blood, metabolomics, placenta, sow

## Abstract

Adding plant extracts to diets to enhance sow performance and health is widely regarded as a healthy and sustainable practice. In promoting antibiotic-free farming, plant extracts have emerged as a leading solution for enhancing sow fertility through nutritional strategies. The aim of this study was to investigate the biochemical impacts of supplementation of sows with ALAEm (composed of nine plant extracts) on blood and placental indices of sows in late gestation. The components of ALAEm were determined by UPLC-MS/MS. 196 normal gestation parturient sows were randomly allocated into two groups (*n* = 98 per group): the control group and the test group fed 20 g/d ALAEm supplementation at 74–114 d of gestation. The study examined the various clinical indexes in the blood, the expression of genes and proteins and metabolomics in the placenta. Dietary ALAEm supplementation improved sow reproductive performance (total number of piglets born alive, number of piglets weaned, wean weight), serum biochemical indices, placental structure and increased gene and protein expression of ZO-1, Claudin-1 and other placental junction-associated factors. ALAEm attenuated placental tissue oxidation, inflammation, and apoptosis, promoted placental growth (EGF and IGF-1) and angiogenesis factors (VEGFA, PIGF and other factors), and increased the nutrient transport in placental (GLUT1 and SNAT2). Dietary ALAEm supplementation decreased the number of metabolites associated with lipid metabolism through alpha-linolenic acid metabolism. Therefore, dietary supplementation of ALAEm in the late gestation may improve fertility by reducing the levels of inflammation, oxidation and apoptosis in placental tissues via the EGFR/VEGFR2-PI3K-AKT1 pathway, promoting placental growth, angiogenesis and nutrient transport, and altering the levels of placental lipid metabolites via *α*-linolenic acid metabolism.

## 1 Introduction

The reproductive performance of sow is influenced by various factors, including breed, feeding practices, environment, reproductive techniques, dietary metabolic levels, and nutrient availability (1). Sows in the late gestation period (74–114 d) require a high level of nutrients to support fetal bone and muscle development. The porcine placenta consists of a maternal part (the maternal vascular endothelial cell layer, uterine connective tissue layer, and endometrial epithelial layer) and a fetal part (the placental chorionic epithelial layer, placental stromal connective tissue layer, and placental vascular endothelial cell layer) and serves as a link between the mother and the fetus (2). The placenta maintains maternal gestation and regulates the growth and development of the fetus, facilitates the exchange of substances and gases, and serves as an immune barrier to safeguard the health of fetal pigs. Nutrients in the maternal circulation accumulate in the placenta through specific transporters. Some of these nutrients are metabolized in the placenta to provide energy for organ maintenance and development or are converted into other active components for the regulation of placental functions (3) and to provide nutrition for fetal development during gestation (4). Thus, placental nutritional regulation is critical for maintaining pregnancy and healthy fetal development (5).

Studies have shown that changes in the uterine environment can disrupt the development and function of the placenta, affecting vascularization (6), structural integrity, and material exchange between the sow and fetus (7). Impaired placental angiogenesis leads to intrauterine growth retardation (IUGR) of the fetal pig and serious pregnancy complications, fetal loss and cessation of gestation (8), ultimately causing a decline in the reproductive performance of the female animal (9–11). Vaginal infection with bacteria in sows during gestation may be caused by poor barn environment, an imbalanced diet, improper pre-breeding practices, and mechanical injuries caused during herd transfer (12, 13). These can lead to inflammation environment, dysregulation of the endometrium and vaginal microbiota and affect reproduction (14). It has been shown that LPS-induced neuroinflammation is associated with the phosphatidylinositol 3-kinase-serine–threonine kinase (PI3K-AKT) pathway (15), which can activate downstream pathways related to oxidation, inflammation, apoptosis, and angiogenesis. To treat reproductive disorders in sows in the late gestation period, nutritional regulation is generally administered, and plant extracts have been used as feed additives. These extracts exhibit homologation, antibacterial, and antioxidant activities, in addition to the capacity to regulate reproductive hormone levels and enhance immunity and production performance (16). In accordance with the theory of traditional Chinese veterinary medicine, the ALAEm is a natural plant complex composed of nine plants: *Angelica sinensis* (Oliv.) Diels, *Glycyrrhiza uralensis* Fisch., *Astragalus membranaceus* (Fisch.) Bge., *Eucommia ulmoides* Oliv., *Atractylodes macrocephala* Koidz., *Populus tomentosa* Carr., *Perilla frutescens* (L.) Britt., Gynostemma pentaphyllum and *Leonurus japonicus* Houtt. (ALAEm). In the preceding research, it was established that the extract mixture of *Angelica sinensis* (Oliv.) Diels, *Glycyrrhiza uralensis* Fisch., *Astragalus mongholicus* (Fisch.) Bge., and *Eucommia ulmoides* Oliv. could enhance the reproductive function of sows (17). In order to provide a higher nutritional value and a more effective formulation, ALAEm was created based on the previous research, adding several plant extracts with antioxidant effects such as Gynostemma pentaphyllum (18) and treating obstructed labour, such as *Leonurus japonicus* Houtt. (19). *Angelica sinensis* (Oliv.) Diels (20), *Glycyrrhiza uralensis* Fisch. (21), *Astragalus mongholicus* (Fisch.) Bge. (22), *Eucommia ulmoides* Oliv. (23), *Atractylodes macrocephala* Koidz (24), and Gynostemma pentaphyllum (25) have the effect of replenishing blood. *Eucommia ulmoides* Oliv., *Atractylodes macrocephala* Koidz, and *Perilla frutescens* (L.) Britt. (26) are known to tranquilize the fetus, and *Leonurus japonicus* Houtt. (27) invigorates blood circulation. *Angelica sinensis* (Oliv.) Diels and *Astragalus mongholicus* (Fisch.) Bge. have anti-inflammatory, immunomodulatory, antioxidant, antiviral, and hepatoprotective effects (22, 28). *Eucommia ulmoides* Oliv. improved the reproductive and lactation performance of sows and promoted the growth of piglets (29).

Under the environment of intensive antimicrobial-free farming, the reproductive performance of sows is facing serious challenges, and the development of new green feed additives can improve the litter size and piglet survival rate of sows, and promote the healthy development of pig farming. The aim of this study was to investigate the mechanism of ALAEm supplementation to improve the reproductive performance of sows in the late gestation period. We hypothesized that ALAEm would cause significant positive changes in the related factors in the blood and placenta of sows, thereby improving the reproductive performance of sows and laying the experimental foundation for the popularization of the use of ALAEm in production.

## 2 Materials and methods

### 2.1 Analysis of extracts

The extracts that compose ALAEm were purchased from Sichuan Hengruitongda Bio-technology Co., China. All the ingredients of ALAEm were in compliance with the National Standard General Requirements for Natural Plant Feed Ingredients of the People’s Republic of China (GB/T 19,424-2018).

ALAEm was subjected to ultra-performance liquid chromatography coupled with tandem mass spectrometry (UHPLC-Q Exactive HFX, Thermo, United States) (30). The analytical conditions were as follows: UPLC: column, Waters HSS T3 (100 × 2.1 mm, 1.8 μm); column temperature, 40°C; flow rate, 0.3 mL/min; injection volume, 2 μL; solvent system, phase A was Milli-Q water (0.1% formic acid), phase B was acetonitrile (0.1% formic acid); gradient program, 0 min phase A/phase B (100:0, v/v), 1 min phase A/phase B (100:0, v/v), 12 min phase A/phase B (5:95, v/v), 13 min phase A/phase B (5:95, v/v), 13.1 min phase A/phase B (100:0, v/v), and 17 min phase A/phase B (100:0, v/v). The ESI source parameters were set as follows: sheath gas pressure, 40 arb; aux gas pressure, 10 arb; spray voltage, +3,000 v/−2,800 v; temperature, 350°C; and ion transport tube temperature, 320°C. The scanning range of the primary mass spectrometry was 70–1,050 Da (scan m/z range), with a primary resolution of 70,000 and secondary resolution of 17,500. The raw data were pre-processed using Progenesis QI software (Waters Corporation, Milford, MA, United States) for baseline filtering, peak identification, peak matching, retention time correction, and peak alignment to obtain a data matrix containing retention time, mass-to-charge ratio, and peak intensity. The peaks containing secondary mass spectrometry data were identified using San Shu Biotechnology’s secondary mass spectrometry database (San Shu Biotechnology Inc., Shanghai, China), and the corresponding cleavage patterns and the metabolites in the biological samples were analyzed qualitatively and quantitatively (31).

### 2.2 Experimental animals, diets, design, and management

The Institutional Animal Care and Use Committee of Guangdong Ocean University determined the guidelines and approved all animal experiments (ethics approval number 2022-scuec-021). All animal experiments were carried out in compliance with the National Research Council’s Guide for the Care and Use of Laboratory Animals. Based on initial body weight, parity, and backfat thickness, 196 gestating sows (Long White × Large White, Body weight of 200 ± 10 kg, Parity of 2, Backfat thickness of 18–22 mm) were randomly allotted to two experimental groups namely; the control and 20 g/d ALAEm supplementation (*n* = 98). The experimental duration was between the 74th d of gestation to farrowing from March to May 2023 at a pig farm in Yulin City, Guangxi Province, China.

The ALAEm group was fed the basal diet supplemented with ALAEm 20 g/d, while the sows in the control group were fed a normal basal diet. The formulation of the experimental diet was conducted in accordance with the nutrient requirements for late-gestation sows as stipulated by the National Research Council (NRC, 2012). Specifically, the ALAEm group followed a dosage regimen based on its classification as a plant-derived feed additive, with optimal dosage determined to be 100 mg/kg–200 mg/kg through comprehensive review of published literature (32–36). Given that the average weight of a sow in the late gestation period (74–114 d) is approximately 200 kg, the calculated dose would be 20–40 g per sow per day. However, due to the cost—optimization, considerations in practical applications, a dose of 20 g/d was selected as the optimal dose. During the trial, sows were fed twice a day (7:30 and 16:00) with free access to water.

### 2.3 Data collection

Birth weight was recorded at birth (Day 0), while within 24 h of parturition, the numbers of total piglets born, live piglets, healthy piglets, weak piglets, stillbirths, and mummified fetuses and the weight of piglets at birth were recorded. Weaning outcomes (number of weaned piglets and their weight) were recorded at 21 days post-farrowing.

Placenta and blood samples were collected from 10 randomly selected sows from each group. Blood samples were collected from the jugular vein in sterile tubes, stored at 37°C for 1 h, centrifuged at 3000 g for 15 min at 4°C, and stored at −80°C until analysis. The 20 sows were closely observed on the day of parturition, and cord blood was collected during parturition to measure lactic acid (LA) and total bile acid (TBA) levels. When the placenta was discharged at the end of parturition, the placental tissue was collected using 75% alcohol for surface disinfection. The whole placental tissue was divided into sampling bags, quickly frozen in liquid nitrogen for quick freezing, and transferred to −80°C for storage until analysis.

### 2.4 Blood components and biochemical indicators

Blood samples were analyzed using a three-category automatic blood cell analyzer (URIT-5180, Guilin Unite Medical Electronics Co., Ltd., China). Biochemical indicators were measured by automatic biochemical analyzers (Chemray 240, 420, 800, Shenzhen Radiometer Life Science and Technology, China). The reagent kits were purchased from Radiometer/Changchun Huili (China), and all steps were performed following the manufacturer’s instructions. Normal ranges were as follows: red blood cell (RBC) (5.0–9.5 × 10^12^/L), white blood cell (WBC) (11.0–22.0 × 10^9^/L), platelet (PLT) (150–700 × 10^9^/L), total protein (TP) (60–80 g/L), albumin (ALB) (34–48 g/L), globulin (GLO) (15–35 g/L), aspartate aminotransferase (AST) (45–125 U/L), alanine aminotransferase (ALT) (5–40 U/L), and alkaline phosphatase (ALP) (45–125 U/L).

### 2.5 Serum analyses

Enzyme-linked immunosorbent assay (ELISA) was used to evaluate IgA (MM-090501), IgM (MM-040201), IgG (MM-040301), IL-6 (MM-041801), ROS (MM-120501), SOD (MM-045001), and porcine progesterone (P)(MM-120501). All kits were purchased from Jiangsu Meimian Industry Co., Ltd., China. The Detection of Lactic Acid Levels in Cord Blood by Lactic Acid (LA) Content Assay Kit (Lot. No. 1012304171) was purchased from Beijing Box Biotechnology Co., Ltd., China. All steps were performed following the manufacturer’s instructions.

### 2.6 Hematoxylin & eosin staining

The placental tissue specimens in paraformaldehyde fixative were dehydrated in gradient ethanol, paraffin embedded after xylene transparency, sectioned, and subjected to hematoxylin (G1001, Servicebio Biotechnology Co., Ltd., Wuhan, China) and eosin (G1004, Servicebio Biotechnology Co., Ltd.) staining. The samples were dehydrated and sealed, and the histologic changes of the placenta were observed by orthopantomography (Sony, Tokyo, Japan).

### 2.7 qPCR assay

Total RNA was extracted from placental tissue samples using TRIzol reagent (TransGen Biotechnology Co., Ltd., China) following the manufacturer’s instructions. The RNA concentration was determined using a NANODROP 2000 (Thermo Fisher Scientific, United States), and the purity of RNA was determined by the A260/A280 ratio. Complementary DNA (cDNA) was synthesized from 1 μg of RNA by reverse transcription using All-in-One-First-Strand Synthesis MasterMix (with dsDNase) (F0202, Beijing Lablead Biotechnology Co., Ltd., China). Real-time fluorescence qPCR of cDNA was performed using Taq SYBR Green qPCR Premix (R0202, Beijing Lablead Biotechnology Co., Ltd.) and DNA-specific primers (Sangon Biotech, Shanghai, China) with *β*-actin (Sangon Biotech) as the reference gene. The primers are listed in Table 1. PCR amplification was performed on the Fluorescent Quantitative Polymerase Chain Reaction (PCR) Detection System (FQD-96X; Hangzhou Bori Technology Co., Ltd., China) following the manufacturer’s instructions. The qPCR conditions were 95°C for 30 s and 40 cycles of 95°C for 10 s and 60°C for 30 s. The melting curve conditions were 95°C for 15 s, 60°C for 1 min, and 95°C for 15 s. Relative mRNA expression was calculated by the 2^-ΔΔCT^ method.

**Table 1:** Primers for real-time quantitative PCR.

Categories	Gene	Primer sequence	Product length	Gene sequence number
Inflammatory factors	IL-6	F:GACGGATGCTTCCAATCTGGGTTC R:TAATCTGCACAGCCTCGACATTTCC	142	NC_010451.4
	IL-10	F:AGCCAGCATTAAGTCTGAGAACAGC R:GGTCAGCAACAAGTCGCCCATC	139	NC_010451.4
	IL-1β	F:TGGTGTCTGTGATTGTGGCAAAGG R:TTTCAAGGACGATGGGCTCTTCTTC	120	NW_018085011.1
	TNF-α	F:GCACTGAGAGCATGATCCGAGAC R:CGACCAGGAGGAAGGAGAAGAGG	120	NC_010449.5
	STAT3	F:TGGAGAAGGACATCAGCGGTAAG R:ACCAGCGGAGACACAAGGATG	142	NC_010454.4
Oxidation factors	SOD	F:GAAGCACAGCCTCCCCGATTTAC R:TCCTGGTACTTCTCCTCAGCGATG	141	NC_010455.5
	CAT	F:GCTGGTTAATGCGAGTGGAGAGG R:AGTCGTGCTGCGTCTTCAACAG	96	NC_010444.4
	HO-1	F:GCACACCCAGGCAGAGAACAC R:GCGGCATAGATGTGGTACAGTGAG	108	NT_176279.1
Apoptosis factors	caspase-3	F:TGTGGGATTGAGACGGACAGTGG R:GCCAGGAATAGTAACCAGGTGCTG	112	NC_010457.5
	BAX	F:CATCTACCAAGAAGTTGAGCGAGTG R:ACGGCTGCGATCATCCTCTG	88	NC_010448.4
	Fasl	F:CCACCAACACTCCTGCCATCAAG R:ATCCCCAGCCCCAATCCAACC	143	NC_010451.4
Angiogenesis-related factors	VEGFA	F:GCCTTGCCTTGCTGCTCTACC R:CAGGACGGCTTGAAGATGTACTCG	103	NC_010449.5
	MMP9	F:GCTCCGACGACATGCTCTGG R:GAATGGAAACACGCAGGGCTTG	131	NC_010459.5
	PPARγ	F:TCTGTGGACCTGTCGGTGATGG R:TCAGCTCTCGGGAATGGGATGTC	122	NC_010455.5
	PIGF	F:TCAAGAGACTGCTGTATGCCCATC R:ACAACCATGTCAAGTGCGTTTCC	117	NC_010445.4
	VEGFR1	F:GGATTCTGCTGCGGACGGTTC R:GAGGCTGAGGGTCACGGAGTAG	98	NC_010453.5
	VEGFR2	F:CCAGATGACAGCCAGACAGACAG R:GGAGCCTTCAGATGCCACAGAC	141	NC_010450.4
Growth and receptor factors	EGF	F:GGTCCTGCTGCTGCTCCTG R:ACTCACATCTCTGCCTGACTCTTC	109	NC_010450.4
	IGF-1	F:CACATCCTCTTCGCATCTCTTCTAC R:GTCTCCGCACACGAACTGAAG	127	NC_010447.5
	EGFR	F:GCCTGGACATAGCGTCCTTG R:CAGATGTGGCCCATGGCTTT	193	NC_010451.4
	IGF1R	F:AGAAGCAGGCAGAGAAGGAGGAG R:CCGTTCAGGTCTGGGCACAAAG	89	NC_010443.5
	TGFβR1	F:TGCCATAACCGTACAGTCATTCACC R:AAGCCTGATCCAGAACCTGATGTTG	137	NC_010443.5
	FGFR1	F:TCCGACAAGGGCAACTACAC R:CTGTCTTGTTGGCTGGCAAC	133	NC_010457.5
	FGFR2	F:CACTGTCCTGCCAAAACAGC R:CAGAATGACCGTCACCACCA	130	NC_010456.5
Tight junction factors	occludin	F:CAGCAGCAGTGGTAACTTGGAG R:CGTCGTGTAGTCTGTCTCGTAATG	109	NC_010458.4
	claudin1	F:ACTCCTACGCTGGTGACAACATTG R:CGACACGCAGGACATCCACAG	71	NC_010455.5
	claudin3	F:TTCATCGGCAGCAGCATTATCAC R:CCAGCAGAGAGTCGTACACTTTG	112	NC_010445.4
	ZO-1	F:CTGACAGTATCCACTCTGCTAATGC R:AGAAGGCTCTGACCGCTGATC	146	NC_010443.4
	TJP1	F:TGATGATCGTCTGTCCTACCTGTC R:CCGCCTTCTGTATCTGTGTCTTC	105	NC_010443.5
	CDH1	F:TCTGCTGCTCCTGCTCCTTATTC R:GTCCTCTTCTCCACCTCCTTCTTC	121	NC_010448.4
	CTNNB1	F:CCATCTGTGCTCTCCGTCATCTG R:GGTAGTCCGTAGTGAAGGCGAAC	85	NC_010455.5
Function-related factors	GLUT1	F:CGCTTCCTGCTCATCAACC R:CCTTCTTCTCCCGCATCAT	136	NC_010448.4
	SNAT1	F: GCAGGTCTTCGGCACCACAG R: GGTAGCTCAGCATTGCTCCAGTG	80	XM_003355629.4
	SNAT2	F: GCCGCAGCCGTAGAAGAATGATG R: AAGCAATTCCGTCTCAACGTGGT	125	NM_001317081.1
	PLET1	F:TCAAGAATCCAGGTCAACCCAAAGG R:TTGCCAGAAGCCAATGACTGAGTG	135	NC_010451.4
	ESR1	F:AGGAACCAGGGCAAGTGTGTC R:CATACGGAAGCGAGATGATGTAGC	78	NC_010443.5
	CCND1	F:GCTCGCCCTCCGTGTCCTAC R:TCGCAGACCTCCAGCATCCAG	94	NC_010444.4
Reference	β-actin	F: GATCTGGCACCACACCTTCTACAAC R: TCATCTTCTCACGGTTGGCTTTGG	107	NC_010445.4

### 2.8 Western blotting

Proteins were extracted from placental tissue with a Protein Extraction Kit (Lot 01408/22,222; Kangwei Century Technology Co., Ltd., China). The bicinchoninic acid (BCA) Protein Assay Kit (Lot 28,523; Kangwei Century Technology Co., Ltd.) was used to assess protein concentrations. Protein samples were separated on 8% sodium dodecyl sulfate-polyacrylamide gels (SDS-PAGE, Lot: P6301010; Yeasen Biotechnology [Shanghai] Co., Ltd. China) and transferred to polyvinylidene difluoride membranes. After blocking in rapid closure solution (Lot: F6310030; Yeasen Biotechnology [Shanghai] Co., Ltd.), the membranes were incubated with primary antibody for 30 min at 4°C. The following primary antibodies were used: VEGFR2 (1:1000, #WL02294), EGFR (1:1000, #WL0682a), Akt1 (1:1000, #WL01652), P-Akt1 (Ser 473) (1:1000, #WLP001), PI3K (1:1000, #WL03380), and eNOS (1:1000, #WL01789), all from Wanleibio Biotechnology Ltd., China. Antibody against Claudin-1 (1:1000, Lot #ab15098) was obtained from Abcam Ltd., United Kingdom. Antibody against P-PI3K (p85α/*β*/p55γ) (1:500, #SC-374534) was from Santa Cruz Biotechnology, United States. Antibody against β-actin (1:1000, #R10602) and horseradish peroxidase-labeled goat anti-mouse IgG (1:1000, #R20619) and goat anti-rabbit IgG (1:1000, #R10327) were from TransGen Biotechnology Co., Ltd., China. Bands were visualized using ECL chemiluminescent solution from Tanon Science & Technology Co., Ltd., China (Cat. No. 180–5001). Blots were scanned using a Tanon infrared fluorescence imaging system (Tanon-5200; Tanon Science & Technology Co., Ltd.). ImageJ software (Imager software, Inc., AL, United States) was used to quantify the protein bands, and normalized protein expression levels were calculated.

### 2.9 Immunofluorescence analysis

Paraffin sections were deparaffinized for antigen repair; after antigen recovery, the samples were blocked in normal serum. Samples were incubated with primary antibodies against ZO-1 (1:200, GB111981) and Claudin1 (1:500, GB15032), both from Servicebio Biotechnology Co. Ltd., overnight at 4°C. The samples were then simultaneously incubated with CY3-labeled goat anti-rabbit IgG (1:300, GB21303) and Alexa Fluor 488-labeled goat anti-mouse IgG (1:400,GB25301) (both purchased from Servicebio Biotechnology Co., Ltd.). DAPI (G1012, Servicebio Biotechnology Co., Ltd.) was used to label the nuclei, followed by treatment with quenching autofluorescence (G1221, Servicebio Biotechnology Co., Ltd.) and antifluorescence-quenching sealer (G1401, Servicebio Biotechnology Co., Ltd.). Images were captured by an upright fluorescence microscope (Nikon, Tokyo, Japan) and processed using ImageJ software (National Institutes of Health, United States).

### 2.10 Non-targeted metabolomics assays

Metabolites were determined in two groups of placenta samples by ultra-performance liquid chromatography coupled with tandem mass spectrometry (UHPLC-Q Exactive HFX, Thermo). The placental tissue was thawed slowly at 4°C, and 50–100 mg was placed into a centrifuge tube. Next, 200 μL of pre-cooled water was added, and the sample was vortexed for 60 s, followed by the addition of 0.8 mL of pre-cooled methanol-acetonitrile mixture (1:1, v/v). The samples were analyzed following the methods described in a previous section. Qualitative, quantitative, and bioinformatics analysis of metabolites was performed in placental tissues by matching information on retention time, molecular mass (molecular mass error within 10 ppm), and secondary fragmentation spectra of metabolites in self-constructed and commercial databases.^1^

### 2.11 Data processing for non-targeted metabolomics

Principal component analysis (PCA) and orthogonal partial least squares discriminant analysis (OPLS-DA) were used to process the UHPLC-Q Exactive data. Differential metabolites were determined by FC > 1.5 or FC < 0.67 with a *p* value < 0.05 and VIP > 1 as the criteria for statistically significant differences. Metabolic pathway enrichment analysis was performed using the integrated HMDB database, KEGG compound database, and LIPID MAPS database.

### 2.12 Statistical analysis

Data were analyzed using SPSS 25 software (IBM Corporation, Armonk, NY, United States) and GraphPad Prism 8.0 software (GraphPad, CA, United States). Numerical value is expressed as means ± standard error. For data that conformed to normal distribution, a two-sided *t*-test was used to compare the means of two groups. The Mann–Whitney U test was used when data did not conform to normal distribution. Correlation was determined using Pearson’s correlation analysis, and a *p* value < 0.05 was deemed to indicate a statistically significant difference. Graphs were prepared using GraphPad Prism 8.0 software.

## 3 Results

### 3.1 Chemical components of ALAEm

**Figure 1:**
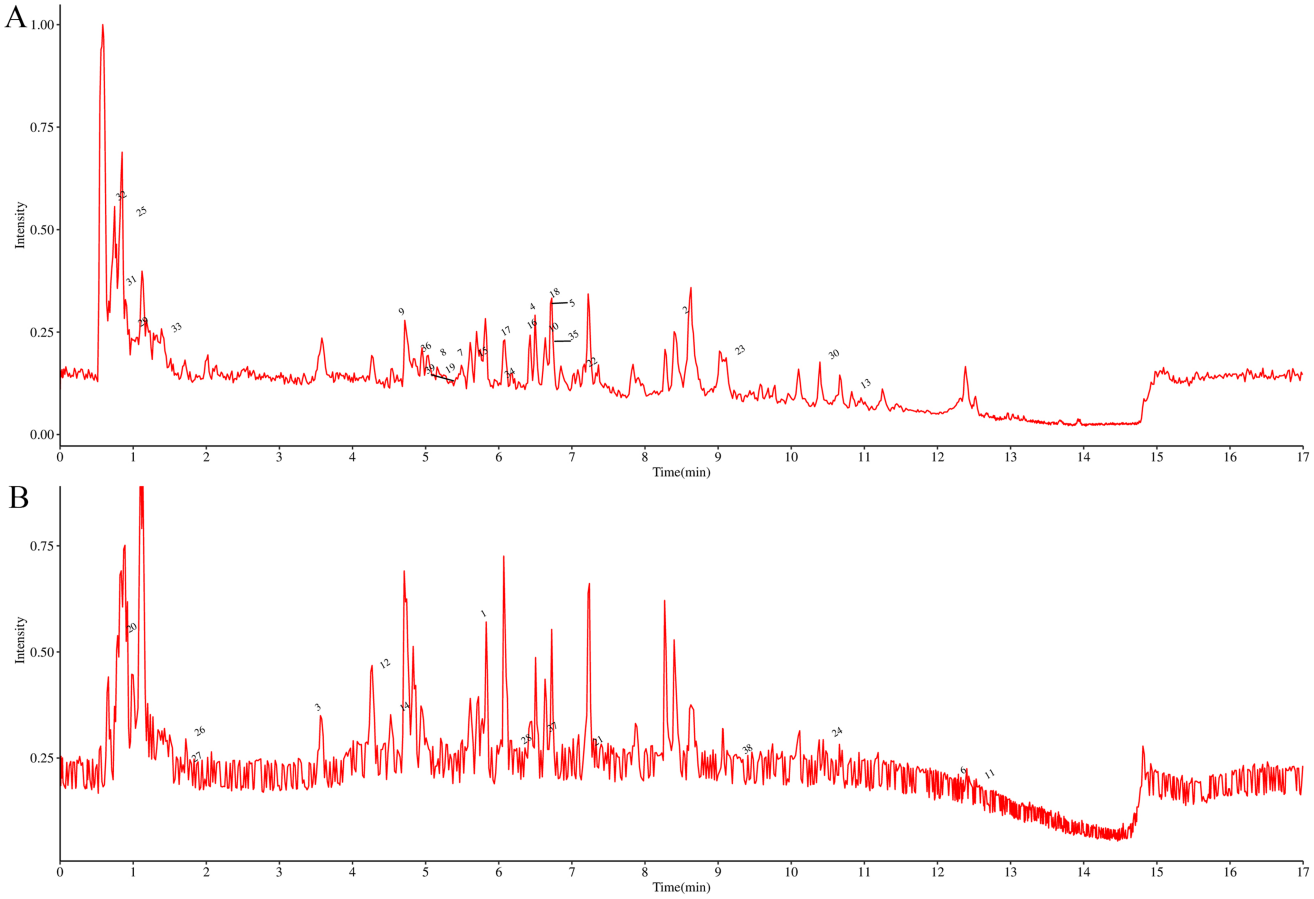
Total ion flow chromatogram of ultra-performance liquid chromatography coupled with tandem mass spectrometry of ALAEm. **(A,B)** Total ion flow chromatogram in positive **(A)** and negative ion **(B)** modes.

The active components in ALAEm were identified using UPLC-MS/MS. A total of 803 active components in ALAEm (Supplementary material 1) were identified by matching with secondary mass spectrometry. All components were analyzed by secondary mass spectrometry. Supplementary material 2 shows the secondary mass spectra of the top 30 components in ALAEm, which were classified into 10 major groups: phenylpropanoids and polyketides, lipids and lipid-like molecules, benzenoids, lignans, neolignans and related compounds, alkaloids and derivatives, organic nitrogen compounds, organic acids and derivatives, organic oxygen compounds, and organoheterocyclic compounds. The total ion flow diagram of ALAEm is shown in Figure 1. Information on the top 20 active ingredients in terms of relative content is shown in Table 2.

**Table 2:** The top 20 active components in ALAEm in terms of relative content.

Number	Name	Class	PubchemID	Formula	m/z	RT (min)	Group area	Adducts
1	Liquiritin	Flavonoids	503,737	C21H22O9	417.119280781248	5.82818333333333	15765556.3901665	M-H, 2 M-H
2	Glycyrrhizic acid	Prenol lipids	14,982	C42H62O16	823.409563180559	8.5933	12856389.8521106	M + H
3	Geniposidic acid	Prenol lipids	443,354	C16H22O10	373.114499971138	3.56058333333333	11406459.7275438	M-H, 2 M-H, M + Cl, M + Na-2H
4	Lamiidoside	Prenol lipids	23,815,404	C26H32O14	551.174912602924	6.49601666666667	10629888.3182296	M + H-H2O
5	Ononin	Isoflavonoids	442,813	C22H22O9	431.132712307734	6.70691666666667	10551070.1587453	M + H
6	Glycyrrhetinate	Prenol lipids	6,857,363	C30H46O4	469.33259627824	12.3872333333333	6318909.85052584	M-H
7	Daidzein-4′,7-diglucoside	Isoflavonoids	171,292	C27H30O14	579.170051005654	5.48851666666667	5770642.93219325	M + H-H2O, M + H, M + H-2H2O
8	Musk ketone	Organooxygen compounds	6,669	C14H18N2O5	312.154494036942	5.01748333333333	5572106.11914579	M + NH4
9	3 Hydroxycoumarin	Coumarins and derivatives	13,650	C9H6O3	163.038631225614	4.71428333333333	5113679.51850721	M + H-H2O, M + H
10	9-Deoxygoniopypyrone	Organooxygen compounds	126,233	C13H14O4	257.080291166898	6.63603333333333	4362775.61593968	M + Na
11	Colubrinic acid	Prenol lipids	21,672,700	C30H46O4	469.332672875787	12.5243833333333	4160198.8858633	M-H, 2 M-H, M + Cl
12	1-Caffeoylquinic acid	Organooxygen compounds	10,155,076	C16H18O9	353.088130555977	4.25008333333333	3906192.45687982	M-H, M + K-2H, M + Na-2H
13	1-Linoleoyl-sn-glycero-3-phosphorylcholine	Glycerophospholipids	11,005,824	C26H50NO7P	520.338940452058	10.8269333333333	3296792.03531289	M + H, M + Na, M + H-H2O
14	Osmanthuside H	Organooxygen compounds	192,437	C19H28O11	431.156274816091	4.52078333333333	3270766.53814627	M-H, M + Cl
15	RUTIN	Flavonoids	5,280,805	C27H30O16	611.159645735714	5.59286666666667	3203725.07177923	M + H
16	Isoliquiritin	Flavonoids	5,318,591	C21H22O9	419.132848014932	6.42846666666667	3144862.99565176	M + H
17	Isoferulic acid	Cinnamic acids and derivatives	736,186	C10H10O4	195.064864268543	6.08091666666667	3063778.09347184	M + H, M + ACN + H, M + H-H2O
18	Formononetin	Isoflavonoids	5,280,378	C16H12O4	269.08024015293	6.70691666666667	3032084.7624773	M + H
19	Vicenin III	Flavonoids	185,958	C26H28O14	565.15436175854	5.22445	2972598.67260747	M + H-H2O, M + H
20	Raffinose	Organooxygen compounds	439,242	C18H32O16	503.161127217343	0.78785	2888583.35021924	M-H

### 3.2 Effect of ALAEm on the reproductive performance of sows

**Figure 2:**
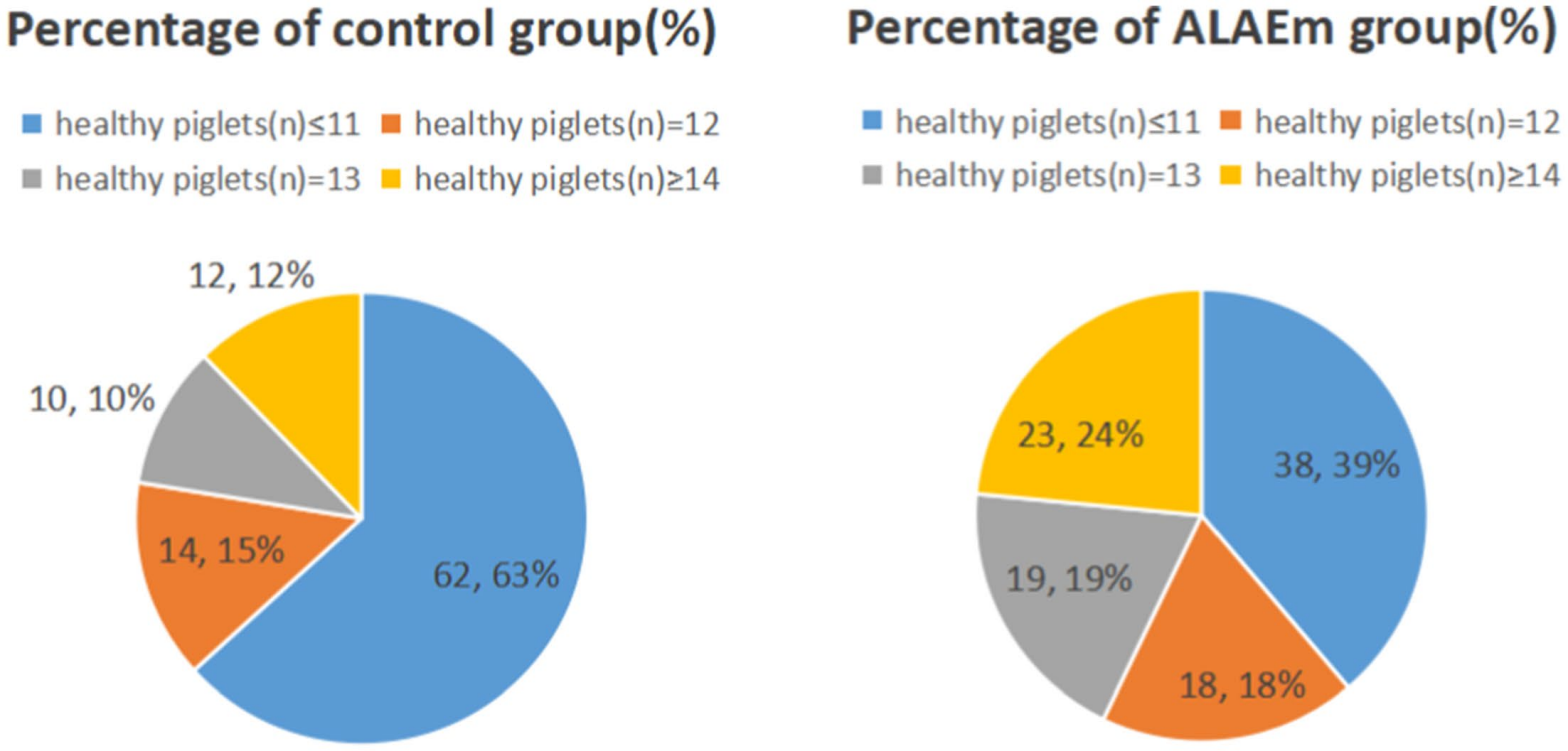
Distribution of healthy piglets as a percentage.

The sows fed dietary ALAEm had significantly higher number of total piglets born alive, healthy piglets, newborn litter weight, number of weaned head, and weaned litter weight at 21 days (*p* ≤ 0.001) (Table 3). Further analysis of the healthy piglets revealed that the percentages of ≥14 and ≤11 piglets in the control group were 12 and 63%, respectively, while the percentages in the ALAEm group were 24 and 39%, respectively, suggesting that ALAEm increased the number of healthy piglets born to low-producing sows (number of healthy piglets ≤11) (Figure 2).

**Table 3:** Effect of ALAEm on the reproductive performance of sows.

Items		Control group	ALAEm group	*p*
The number of (*n*)	Total piglets born	11.32 ± 0.31	13.06 ± 0.31	<0.001
	Live piglets born	10.42 ± 0.32	11.96 ± 0.28	0.001
	Healthy piglets^a^	10.13 ± 0.33	11.74 ± 0.28	<0.001
	Weak piglets	0.29 ± 0.07	0.21 ± 0.06	0.517
	Stillbirths	0.46 ± 0.1	0.44 ± 0.09	0.595
	Mummies	0.18 ± 0.06	0.46 ± 0.09	0.005
The weight of (kg)	Piglets weight at birth	15.86 ± 0.50	18.13 ± 0.40	0.001
The number of (*n*)	Weaned piglets	10.38 ± 0.2	11.59 ± 0.15	<0.001
The weight of (kg)	Litter weight at weaning	76.72 ± 1.42	83.77 ± 0.93	<0.001

Data are shown as mean ± standard error (*n* = 98).

a Healthy piglets were defined as newborn piglets weighing ≥0.75 kg.

### 3.3 Effect of ALAEm on the levels of factors in sow serum and cord blood

**Figure 3:**
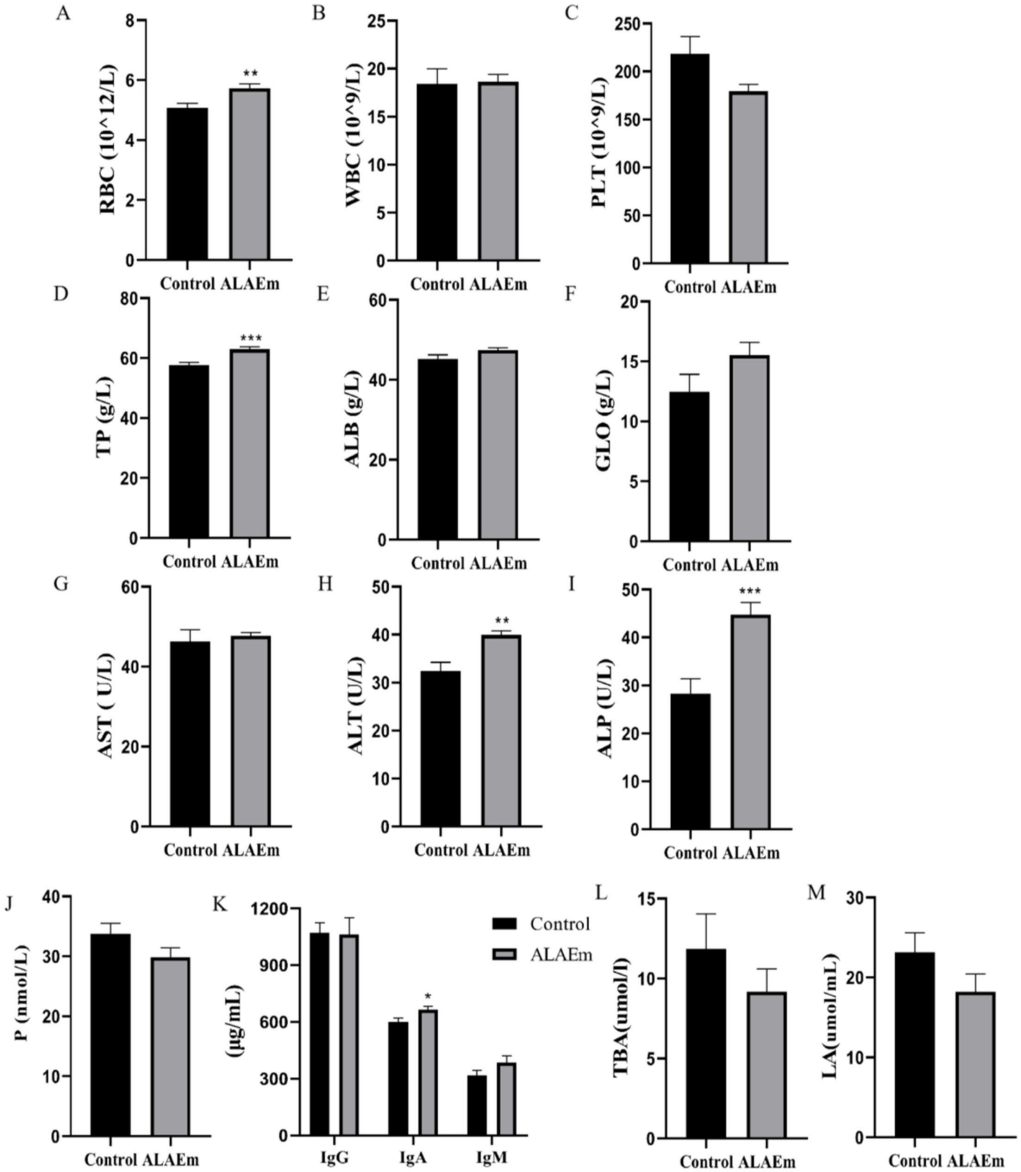
Effect of ALAEm on factors in sow serum and umbilical cord blood. **(A)** Red blood cell (RBC). **(B)** White blood cell (WBC). **(C)** Platelet (PLT). **(D)** Total protein (TP). **(E)** Albumin (ALB). **(F)** Globulin (GLO). **(G)** Aspartate aminotransferase (AST). **(H)**Alanine aminotransferase (ALT). **(I)** Alkaline phosphatase (ALP). **(J)** Progesterone (P). **(K)** IgG, IgA, and IgM. **(L)** Total bile acid (TBA). **(M)** lactic acid (LA). * *p* < 0.05; ** *p* < 0.01; *** *p* < 0.001.

RBC, WBC, and PLT counts were within the normal range in the control and ALAEm groups; however, the RB counts were significantly higher in the ALAEm compared with the control group (*p* < 0.01) (Figure 3A). ALT (*p* < 0.01), TP and ALP (*p* < 0.001) were highly significant increased (Figures 3D,H–I). IgA levels increased significantly after ALAEm treatment (*p* < 0.05) (Figure 3K). There were no significant differences in the other factors (Figure 3).

### 3.4 Effect of ALAEm on the levels of placental tissue factors in sows

#### 3.4.1 Placental connection function

##### 3.4.1.1 Structure of the placenta

**Figure 4:**
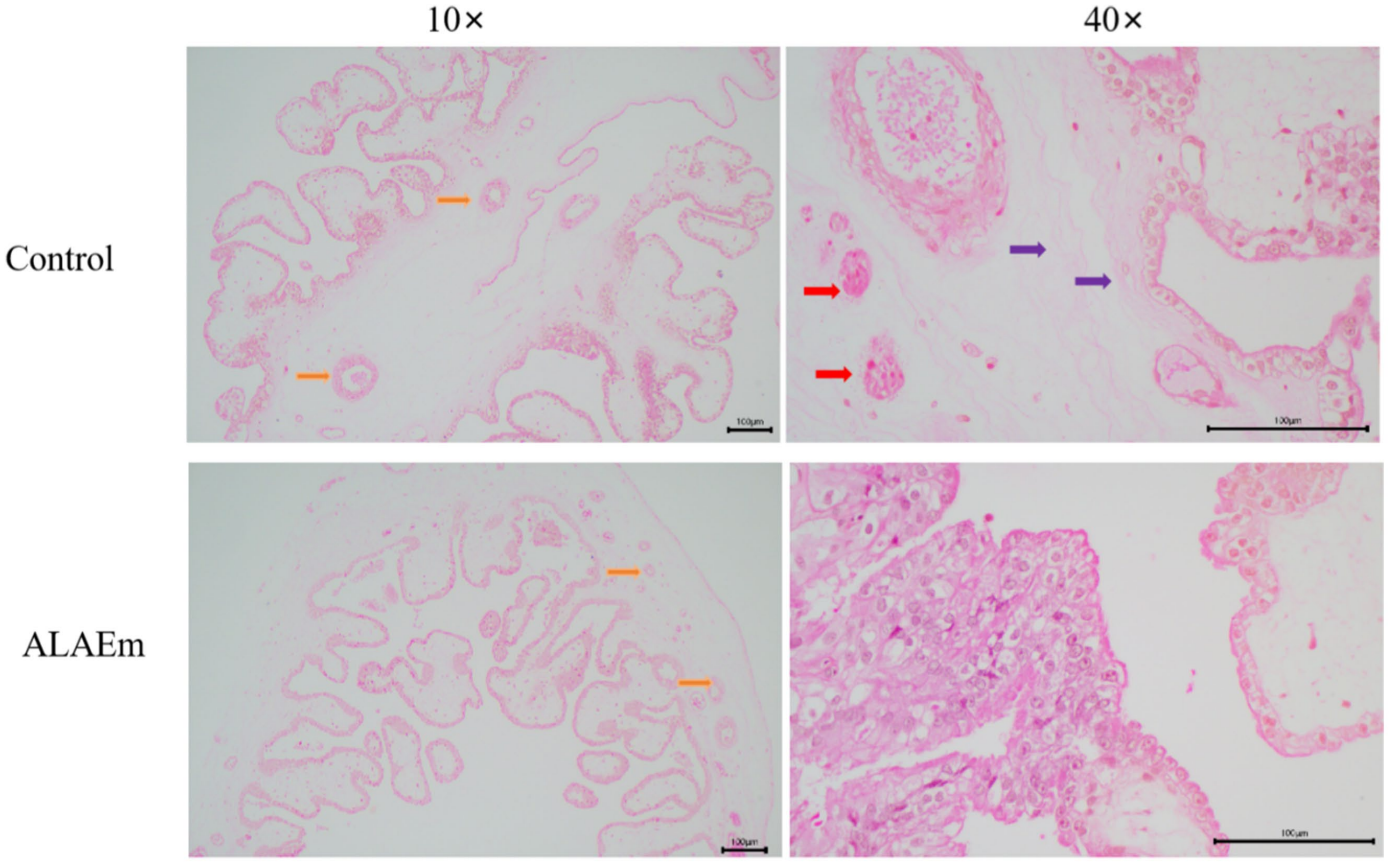
Effect of ALAEm on the placental structure. Yellow arrows: chorionic vasculature; red arrows: syncytiotrophoblast nodules; purple arrows: chorionic interstitial fibrosis.

Hematoxylin & eosin staining of longitudinal section of placenta showed that the placenta of both groups had a generally normal structure; however, because of multiple births, the placenta showed intervillous fibrosis and fibrinoid necrosis of chorionic interstitium, nodularity of syncytiotrophoblasts, and reduction of chorionic vasculature. In the ALAEm group, the structure of the placenta was improved, and the chorionic barrier was intact without obvious edema and abnormal fibrosis (Figure 4).

##### 3.4.1.2 Placental connection factors

**Figure 5:**
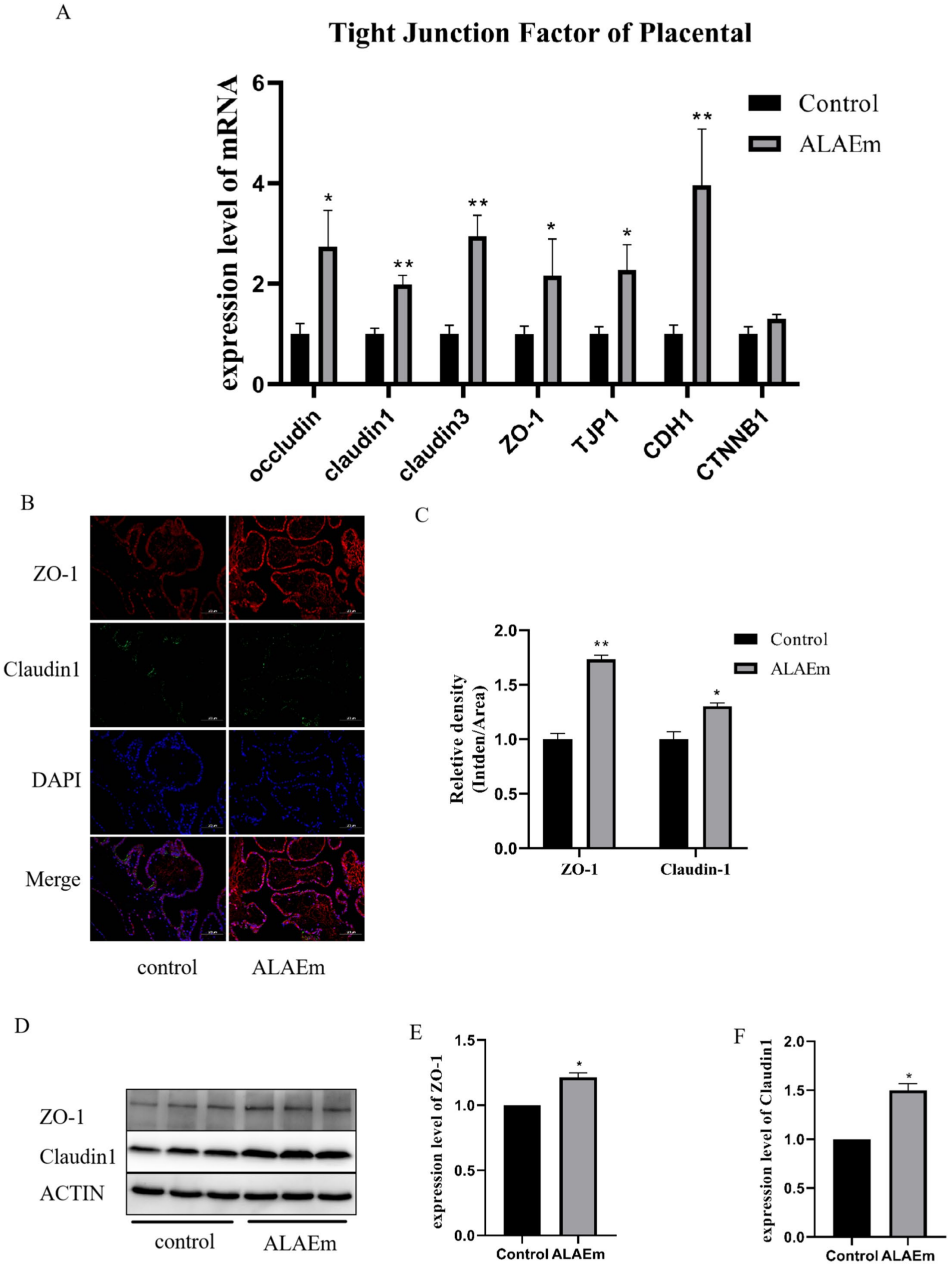
Effect of ALAEm on placental tight junction factors. **(A)** mRNA expression levels of tight junction factors in placental tissues of control and ALAEm. **(B)** Immunofluorescence analysis of ZO-1 and claudin1 levels in placental tissues. **(C)** Statistical analysis of ZO-1 and claudin1 levels. **(D)** Western blotting of ZO-1 and claudin1. **(E,F)** Quantification of protein expression levels of claudin1 **(E)** and ZO-1 **(F)** in placental tissue.

Tight junction-associated factors are expressed in placental tissue. Abnormalities in these factors can affect endometrial tolerance, placental implantation, and corpus luteum function during pregnancy (37). Tight junction-associated factors are involved in the physiological processes and pathology of pregnancy and can be used as markers of uterine tolerance (38). The mRNA levels of tight junction factors in placental tissues were detected using qPCR assay; the expression levels of claudin1, claudin3, CDH1 (*p* < 0.01), occludin, ZO-1 and TJP1 (*p* < 0.05) mRNAs were significantly elevated in the ALAEm group compared with the control group; there was a tendency for CTNNB1 mRNA to be elevated, but not significant (*p* > 0.05) (Figure 5A). The expressions of ZO-1 and claudin1 proteins in placental tissues were examined using immunofluorescence and western blotting. Both proteins were detected in placental tissues, and the expression levels of ZO-1 and claudin1 were significantly elevated in the ALAEm treatment group (*p* < 0.05) (Figures 5B–F).

#### 3.4.2 Oxidation-, inflammation- and apoptosis-related factors

**Figure 6:**
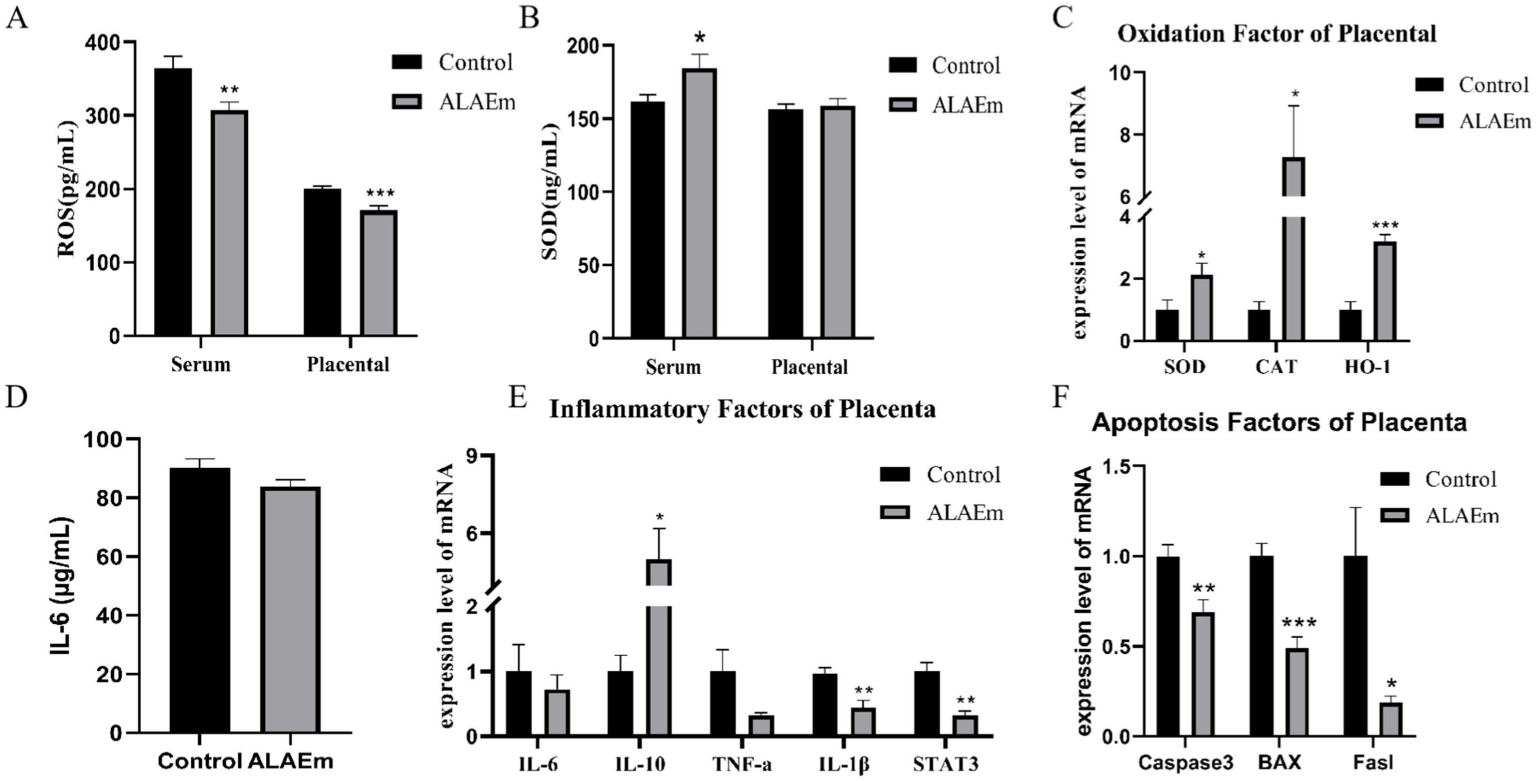
Effect of ALAEm on oxidation, inflammation, and apoptosis factors in placenta. **(A,B)** The content of ROS **(A)** and SOD **(B)** in serum and placenta. **(C)** mRNA expression levels of oxidation factors. **(D)** IL-6 levels in serum. **(E,F)** mRNA expression levels of inflammatory **(E)** and apoptosis factors **(F)** in placenta.

Oxidation levels in serum and placental tissues of sows were detected using ELISA. ALAEm highly significantly reduced ROS levels in serum and placental tissues of sows (*p* < 0.01) (Figure 6A) and significantly increased serum levels of the antioxidant enzyme SOD (*p* < 0.05) (Figure 6B). The mRNA expression levels of antioxidant enzymes SOD, CAT and HO-1 in placental tissues were examined using qPCR assay; all mRNA levels were all significantly elevated (*p* < 0.05) (Figure 6C). ELISA showed that ALAEm decreased IL-6 levels (*p* > 0.5) (Figure 6D).

The mRNA expression levels of inflammation-related factors in placental tissues were detected by qPCR assay. ALAEm treatment significantly increased the mRNA expression of the anti-inflammatory factor IL-10 (*p* < 0.05) and highly significantly decreased the mRNA expression of the pro-inflammatory factor IL-1β and STAT3, which promotes the infiltration of inflammatory cells (*p* < 0.01). There were no significant differences in the other factors (Figure 6E).

ALAEm significantly reduced the mRNA expression levels of the apoptosis-associated factors Caspase-3 (*p* < 0.05), Bax (*p* < 0.001), and Fasl (*p* < 0.05) in placental tissues (Figure 6F).

#### 3.4.3 Placental growth, angiogenesis-related factors and other factors

**Figure 7:**
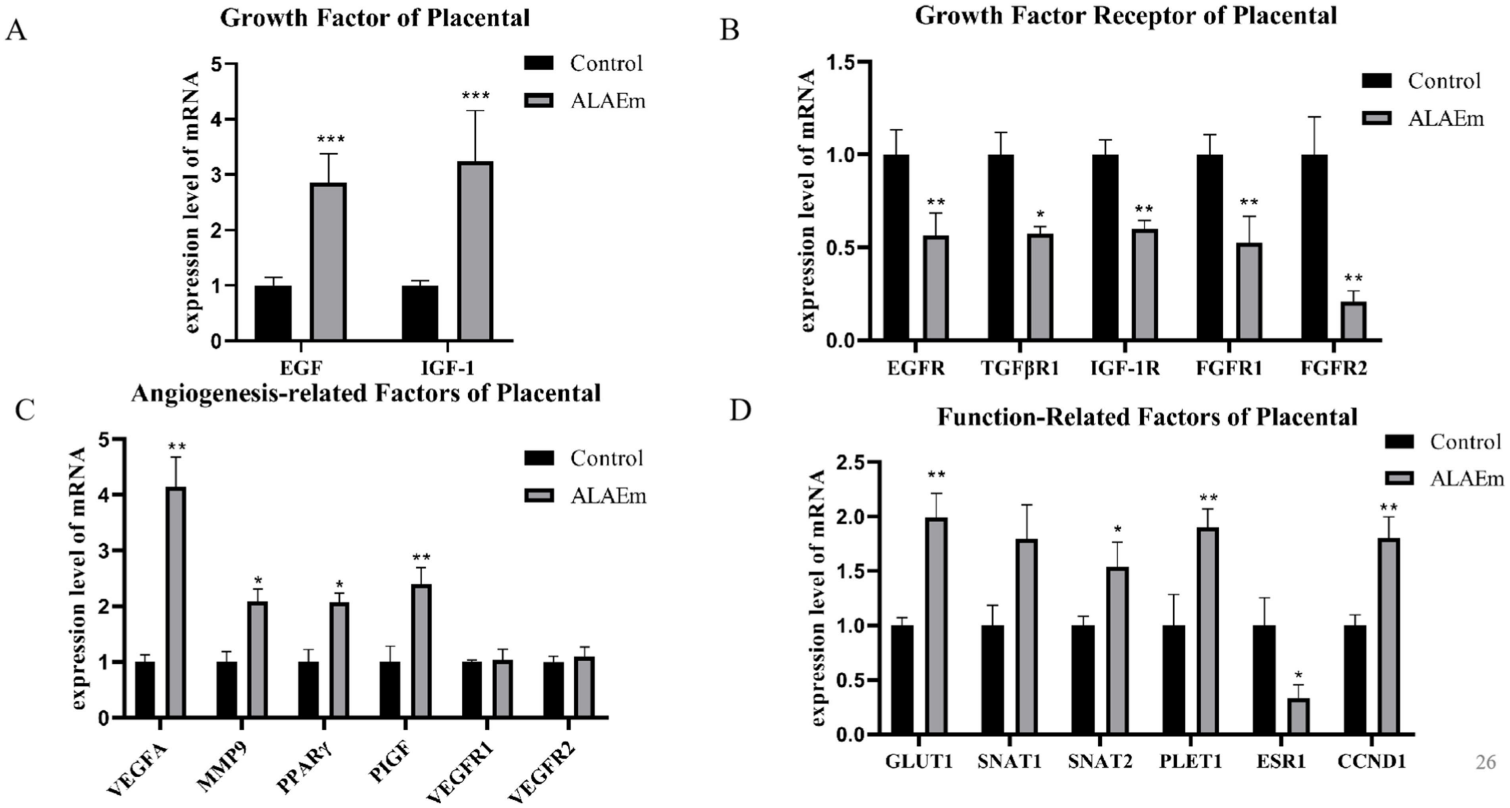
Effect of ALAEm on the levels of growth-related factors, angiogenesis and other factors in placenta. **(A–D)** Levels of mRNA expression of the growth-related **(A)**, growth receptor **(B)**, angiogenesis-related **(C)**, and other factors **(D)**.

Examination of the mRNA expression of growth factors and their receptors in placental tissues by qPCR assay revealed a highly significant increase in the expression of EGF and IGF-1 mRNAs in the ALAEm group (*p* < 0.01) (Figure 7A) and a highly significant decrease in the mRNA expression of growth factor TGFβR1 (*p* < 0.05), EGFR IGF1R FGFR1 and FGFR2 (*p* < 0.01) (Figure 7B). Evaluation of angiogenesis-related factors in the placental tissues of sows showed that ALAEm treatment highly significantly increased the mRNA expression of VEGFA and PIGF (*p* < 0.01) and significantly increased the mRNA expression of MMP9 and PPARγ (*p* < 0.05). There were no significant differences in the other factors (Figure 7C).

In the late gestation period, placental nutrient transport is critical, and the expression of nutrient transport-related proteins, such as GLUT1, SNAT1, and SNAT2, which affects nutrient transfer between the mother and fetus, leading to pregnancy outcome. Placenta expressed transcript 1 (PLET1) may affect the interaction of trophoblast cells with maternal endometrial cells and be associated with placental development (39). Targeting CCDN1 to CCND1 blocks cell cycle progression and promotes apoptosis in uterine smooth muscle tumor cells (40). ESR1, which encodes an estrogen receptor implicated in the development of endometrial cancer, has been linked to recurrent miscarriages (41). The expression levels of SNAT2 (*p* < 0.05), GLUT1, PLET1 and CCND1 genes were highly significantly increased (*p* < 0.01) and the expression of the ESR1 gene was significant decreased (*p* < 0.05) after ALAEm treatment (Figure 7D).

### 3.5 Mechanistic studies on the effect of ALAEm on sow reproduction

**Figure 8:**
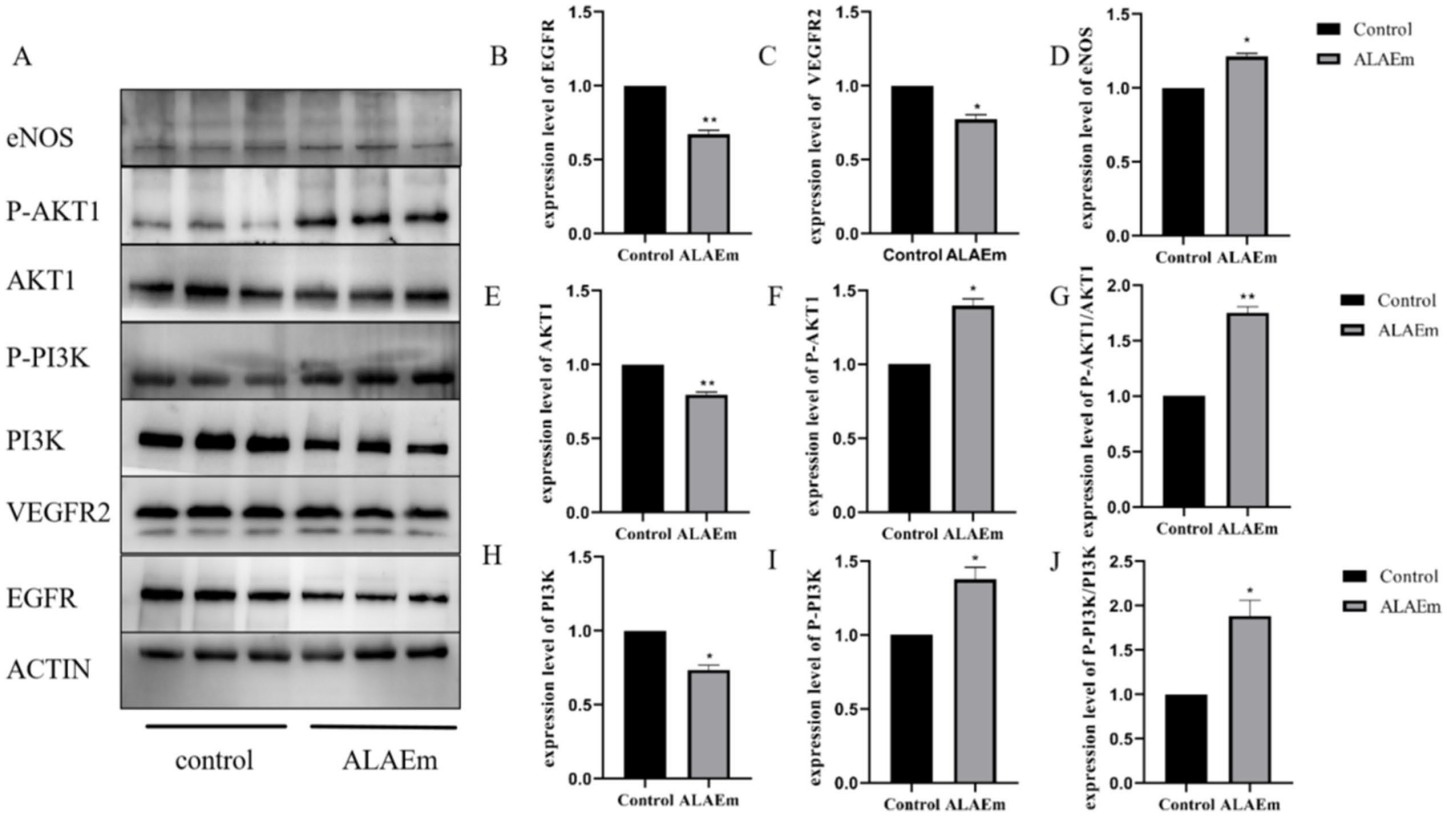
Effect of ALAEm on the expression levels of key proteins in the EGFR/VEGFR2-PI3K-AKT1-eNOS signaling pathway in placenta. **(A)** Western blotting of EGFR, VEGFR2, eNOS, and the unphosphorylated and phosphorylated (P) forms of PI3K and AKT1 after treatment with ALAEm. **(B–J)** Protein expression levels of EGFR, VEGFR2, eNOS, AKT1, P-AKT1, PI3K, and P-PI3K and ratios of P-AKT1/AKT1 and P-PI3K/PI3K after treatment with ALAEm.

Compared with the control group, the ALAEm treatment group showed significantly decreased protein expression of EGFR (*p* < 0.01), VEGFR2 (*p* < 0.05), AKT1 (*p* < 0.01) and PI3K (*p* < 0.05) (Figures 8B,C,E,H), significantly increased expression of P-PI3K, P-AKT1 and eNOS (*p* < 0.05) (Figures 8D,F,I), and significantly increased ratio of P-AKT1/AKT1 (*p* < 0.01) and P-PI3K/PI3K (*p* < 0.05) (Figures 8G,J). ALAEm regulation of sow reproduction may be accomplished by modulating the expression levels of key proteins in the EGFR/VEGFR2-PI3K-AKT1-eNOS signaling pathway in placental tissues (Figure 8A).

### 3.6 Metabolomics profiling

**Figure 9:**
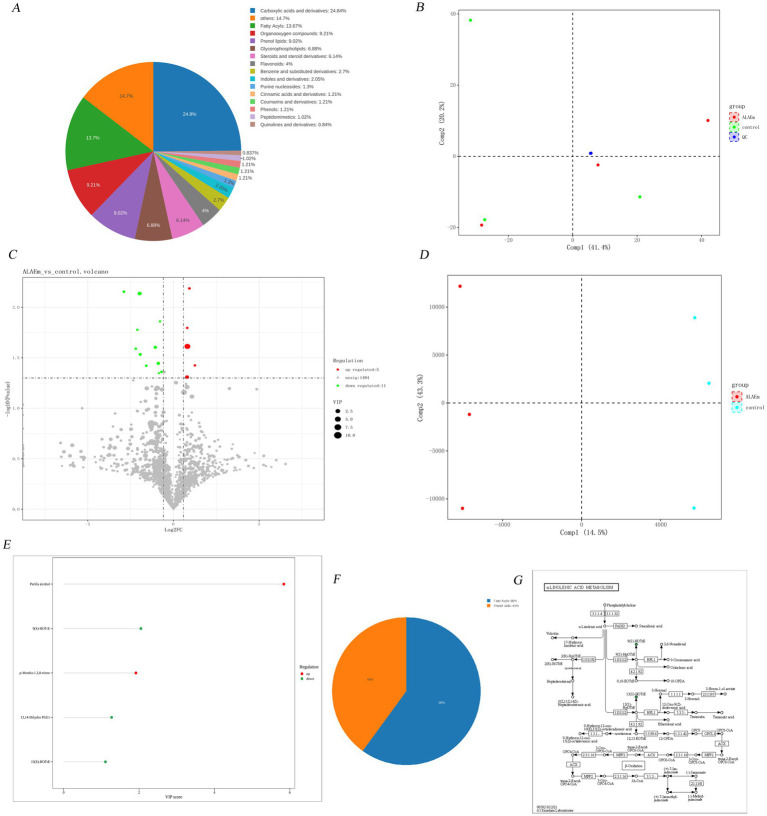
Metabolome profiling. **(A)** Chemical taxonomy. **(B)** PCA score plot. **(C)** Volcano plots. The color of the dots indicates the changes in metabolite content: red indicates upregulated, green indicates downregulated, and gray indicates no significant difference. The size of the dot indicates the size of the metabolite VIP value. **(D)** OPLS-DA score plots. The horizontal coordinate Comp1 represents the first principal component, the vertical coordinate Comp2 represents the second principal component, and the numerical percentage in parentheses represents the variance explained for the principal component. **(E)** VipScore plot. **(F)** Pie class plot. **(G)** KEGG annotation plot. Green indicates a relative downregulation.

A total of 1,532 metabolites were identified by categorizing all metabolites in the control and ALAEm groups (Supplementary material 3). The pie chart statistics of the number of metabolites in each category as a proportion of the number of all metabolites were presented as class B classification results (Figure 9A). As shown in the PCA score plot (Figure 9B), there was a clear separation between the control and ALAEm groups, indicating that the QC samples were highly aggregated, the sample reproducibility was good, the analytical system was stable, and the obtained data were reliable. Fold change (FC) is the ratio of the mean of the quantitative values of all the biological replicates of each metabolite in a comparison group. A metabolite was considered to be a differential metabolite in the volcano diagram (shown in Figure 9C) when FC > 1.5 or FC < 0.67 and *p* value <0.05. A total of 16 differential metabolites were identified and are listed in Table 4.

**Table 4:** Differential metabolites.

Metabolite	A_mean	B_mean	FC	VIP	*p* value	Sig
13,14-Dihydro PGE1	119213.430	220377.433	0.541	1.273	0.036	Down
Linalool 3,7-oxide beta-primeveroside	2689.356	12358.119	0.218	0.404	0.026	
9(S)-HOTrE	79602.477	312607.963	0.255	2.049	0.007	Down
Homovanillic acid	30895.766	16219.664	1.905	0.508	0.007	
3-Aminoisobutanoic acid	18590.638	10649.391	1.746	0.373	0.016	
Ribose 1-phosphate	16241.223	62191.018	0.261	0.880	0.029	
4-Hydroxystyrene	5122.814	8818.327	0.581	0.253	0.014	
Austalide K	1444.794	6236.306	0.232	0.290	0.017	
Leu Pro	2603.309	19385.169	0.134	0.554	0.007	
Perilla alcohol	4904323.917	2791217.682	1.757	5.839	0.024	Up
p-Mentha-1,3,8-triene	580857.325	335002.143	1.734	1.916	0.049	Up
Ile Val Gly	7456.669	22389.734	0.333	0.476	0.038	
13(S)-HOTrE	68676.791	142863.589	0.481	1.109	0.025	Down
6-Ketoestriol	15386.800	6470.896	2.378	0.378	0.038	
Anhydroretinol	27519.702	44678.996	0.616	0.527	0.044	
SCLAREOLIDE	1986.008	3589.731	0.553	0.159	0.045	

R^2^Y(cum) and Q^2^(cum) are the parameters for evaluating the goodness of the model; generally, when Q^2^(cum) > 0.5, the OPLS-DA model is stable and reliable with good fitting and prediction ability. Further analysis using OPLS-DA revealed a marked difference in endogenous metabolites between the two groups (Figure 9D). Potential biomarkers were screened by the VIP value in the VIP value graph (Figure 9E); biomarkers with a VIP value greater than 1 were considered qualified. *T*-test combined with multivariate analysis of OPLS-DA was used to add the condition of VIP > 1 to the screening results of differential metabolites; the classification pie charts are shown in Figure 9F, and the information of the substances is shown in Table 5. Five differential metabolites were screened (three down-regulated and two up-regulated), belonging to fatty acids and acrylolipids; box line diagrams of the five substances are shown in Supplementary material 4.

**Table 5:** Classification of the differential metabolites.

Metabolite	classA	classB	classC	Sig
13,14-Dihydro PGE1	Lipids and lipid-like molecules	Fatty Acyls	Eicosanoids	Down
9(S)-HOTrE	Lipids and lipid-like molecules	Fatty Acyls	Lineolic acids and derivatives	Down
Perilla alcohol	Lipids and lipid-like molecules	Prenol lipids	Monoterpenoids	Up
p-Mentha-1,3,8-triene	Lipids and lipid-like molecules	Prenol lipids	Monoterpenoids	Up
13(S)-HOTrE	Lipids and lipid-like molecules	Fatty Acyls	Lineolic acids and derivatives	Down

The KEGG ID numbers of the differential metabolites were obtained from the database. The metabolic pathway maps obtained from the pathway annotation analysis of the differential metabolites through the KEGG database were enriched to the alpha-linolenic acid metabolism pathway (Figure 9G), which was consistent with the relatively high content of lipid active ingredients in ALAEm. This indicates that the regulation of sow reproduction may be accomplished by lipids through alpha-linolenic acid metabolism.

## 4 Discussion

In this study, the main active components of ALAEm were determined by UPLC-MS/MS. Among the top 20 active ingredients in terms of relative content, nine were classified as phenylpropanoids and polyketides, which were further classified as flavonoids and isoflavonoids. Liquiritin, a flavonoid, was also identified and the highest relative content was observed for this component. Subsequently, a higher relative content of prenol lipids and Fatty Acyls belonging to the lipids and lipid-like molecules classes was noted, including Glycyrrhizic acid, Geniposidic acid, and alpha-linolenic acid. Therefore, we hypothesize that the regulation of sow reproductive performance may be mainly related to the flavonoids and lipids in ALAEm.

Our results also showed ALAEm modulation of sow reproductive performance by increasing the number of weaned piglets and average weaning weight. ALAEm has been found to improve placental status, facilitating increased nutrient delivery to the fetal pig and resulting in increased piglet birth weight. This is achieved by regulating various factors including sow serum factors, placental structure, growth, and angiogenesis, which in turn results in higher weaned piglet weights. Studies have shown that there is an important relationship between piglet birth weight and pre-weaning mortality, and that the pre-weaning mortality rate of low-birth-weight piglets was 34.4%, which accounted for 43% of the total pre-weaning mortality (42), therefore ALAEm also promoted the weaning weight of piglets. *Angelica sinensis* (Oliv.) Diels, *Glycyrrhiza uralensis* Fisch., *Eucommia ulmoides* Oliv. and *Astragalus membranaceus* (Fisch.) Bge., as the main components of ALAEm, have important roles in regulating reproduction. Adding plant extracts during gestation can improve sow reproduction indexes, mainly by improving the inflammatory response and hormone levels and regulating placental function (43, 44). For example, feeding 2% Radix Astragali Extracts compound preparation every day from 70 d of gestation to 21 d of weaning can regulate the state of sows before and after farrowing, which plays a role in increasing the total protein, albumin content, and estradiol level in the plasma of the sows and the immunoglobulin content in the serum and colostrum and decreasing the level of progesterone (45). The herb combination Dang Gui-Yi Mu improved reproductive performance of sows by preventing abortion through regulating T helper (Th)1/Th2 cells (46).

The umbilical cord, as one of the key organs connecting the sow and the fetus during late pregnancy, affects the outcome of piglets through the maternal—fetal integrated system. Sows are prone to LA and TBA metabolic abnormalities during late pregnancy. Some studies have found that piglets born in the posterior quarter are more likely to develop lactic acidemia and are more likely to be stillborn (47). Sows with significantly elevated TBA levels in late pregnancy compared to mid—pregnancy had a stillbirth rate as high as 76.2%, and the performance of piglets after birth was also reduced to varying degrees (48). This abnormality is mainly caused by an oxidative stress response due to liver injury and can lead to decreased fetal piglet survival (49) and altered newborn piglet survival and litter weight at weaning (50). We found that ALAEm treatment reduced LA and TBA levels in cord blood, increased piglet survival, decreased ROS accumulation, and increased antioxidant enzyme levels in the serum and placental tissues of sows with elevated oxidative levels. Many blood indices and biochemical indices change during the gestation of sows, and blood volume increases gradually during gestation until the end of gestation. Thus, the effect of ALAEm on blood indices in sows was also investigated in this study. We found that ALAEm had the effect of reducing anemia and platelet accumulation, which may be through its regulation of PI3K-AKT1 signaling and the subsequent anti-thrombosis effects (51). ALAEm increased the levels of TP, AST, ALT, and ALP, reflecting improved hepatic synthesis and reduced occurrence of protein loss because of renal pathology. ALAEm also significantly elevated IgA levels, which may represent enhanced mucosal immunity, indicating improved immune function of the experimental sows. ALAEm shows a potential tendency to shorten the days to return to estrus after weaning as inferred from the changes in progesterone levels (52, 53). Therefore, ALAEm improves sow reproduction by affecting the levels of related factors in sow serum and cord blood.

In epithelial cells, tight junctions (TJs) and adhesion junctions (AJs) integrate to form a protein complex called the cell adhesion apical junctional complex (AJc), which constitutes the placental barrier. Tight junction structures and tight junction-associated factors (Claudin, Occludin, ZO-1, TJP1) regulate biological processes during placenta formation that affect endometrial tolerance and placental implantation during pregnancy (54). Reduced CTNNB1 expression is observed in chorionic and fetal membranous tissues in recurrent miscarriages (55) and inhibits trophoblast cell proliferation and invasion (56); studies showed an involvement of ZO-1 protein in trophoblast cell differentiation (57) and reduction of occludin and claudin1 expression in the placenta in pre-eclampsia (58). We infer that ALAEm may potentially play a role in promoting trophoblast cell functions similar to the beneficial effects associated with higher levels of these factors. However, further functional studies are needed to confirm this speculation. ALAEm treatment increased the expression of TJ and AJ-related factors in placental tissues, thereby improving placental structure and strengthening the placental barrier. Placental structure directly affects placental angiogenesis and placental growth, and factors regulating placental angiogenesis mainly include PPARγ, MMP9, and PIGF. PPARγ affects trophoblast invasion and placental angiogenesis by regulating VEGF (59, 60) and regulates progesterone content (61); its deficiency leads to placental abnormalities and fetal death (62). MMP9 plays a role in angiogenesis and remodeling (63) and is downregulated in the placenta in IUGR (64). PIGF is involved in the activation of STAT3 signaling, which is closely related to angiogenesis (65). EGF and IGF-1 are key factors that promote placental growth, and the interaction of EGF and EGFR activates the tyrosine-protein kinase C system, which is involved in placental trophoblast cell infiltration, proliferation, differentiation, and endocrine regulation. EGFR is also associated with the growth of tumor cells and functions as an immune-suppressor factor to promote cervical tumor growth through the STAT3 pathway (66). The vivo increase in IGF-1 concentration may enhance placental nutrient transport, thereby stimulating fetal IGF-1 production and further promoting fetal growth (67). ALAEm treatment increased the expression of VEGFA, PPARγ, MMP9, PIGF, and EGF and decreased the expression of their receptors in placental tissues. So, it is inferred that the improvement of placental connectivity, growth and angiogenesis may be the key to the regulation of placental function by ALAEm in sows.

Abnormalities in placental structure lead to a reduced exchange area, limiting nutrient uptake and transport, which becomes the morphological basis that leads to placental hypofunction, ultimately leading to fetal growth restriction, the production of weak or stillborn piglets, and an impact on the body weight of weaned piglets (5, 68). GLUT1, SNAT1, and SNAT2 are responsible for the transport of glucose and neutral amino acids (e.g., glutamine, alanine, and proline) from the sow to the fetus (69). The addition of ALAEm caused an increase in the gene expression levels of all three nutrient transporters in the placenta, which may facilitate the supply of nutrients from the sow to the fetus, and may also be related to the improvement in placental structure observed in histomorphometric observations. PLET1 expression is upregulated with the progression of pregnancy (70). Upregulated ESR1 was associated with recurrent miscarriages (41). Our results suggest that ALAEm treatment increased PLET1 and decreased ESR1 expression, which may promote placental development nutrient transport and reduce the occurrence of recurrent miscarriage. In this paper, the newborn weight of fetal pigs in the ALAEm group was significantly higher than that of the control group, which could may be attributed to the improvement of placental structure and nutrient transportation function.

Incorrect husbandry and management practices in the second trimester of gestation and multiple deliveries can lead to changes in the sow uterus, and accumulation of microbial products and host inflammatory mediators (e.g., cytokines and chemokines) can lead to apoptosis of the trophoblast cells, resulting in the expulsion of the embryo or fetus (71). In late gestation, rapid fetal development, increased energy intake of sows, and accelerated maternal metabolism all lead to the increased production of ROS (72). Accumulation of ROS passes through the placenta and causes fetal death (73) and the development of pregnancy complications, such as IUGR (74), which ultimately impairs fertility. In this paper, ALAEm treatment reduced serum and placental tissue levels of inflammatory factors IL-6, IL-1β, and TNF-*α*, increases levels of the proinflammatory factor IL-10, reduces ROS levels, and increases antioxidant enzyme activity. VEGFA inhibits ROS production and downregulates the NF-kB pathway to exert an anti-inflammatory effect (75); it also inhibits uterine necrosis and apoptosis (62). Flavonoids are indeed the primary bioactive components in ALAEm. Studies have shown that flavonoids can inhibit apoptosis in mitochondria and improve the function of the antioxidant system by enhancing SOD activity and scavenging ROS (76, 77), and also have hepatoprotective effects (78). For example, Daidzein is an isoflavonoid that improves embryo survival by enhancing the levels of estrogen and GSH-Px in the amniotic fluid of sows and participating in the arginine and proline metabolic pathways (35). Quercetin is another flavonoid that reduces P release and promotes IGF-1 secretion (79). So, it is inferred that the relatively high content of flavonoids in ALAEm may be a key factor influencing inflammation, oxidation, and apoptosis in placental tissues. VEGFA is a pro-angiogenic active factor that stimulates placental angiogenesis and growth, resulting in a greater flow of nutrients from the sow to the fetus and improvement in fetal growth needs (80). When VEGFA binds to VEGFR2, it blocks VEGF-induced AKT phosphorylation and inhibits endothelial cell migration, invasion, conduit formation, and angiogenesis (81) and activates STAT3 to achieve proliferative and antimodulatory cell signaling (82). Activation of the STAT3/VEGFA pathway enhances angiogenesis (83), and STAT3 is significantly upregulated in preeclamptic placenta (84). We previously demonstrated that EGFR-PI3K-AKT1 is a key regulatory pathway mediated by the main component of ALAEm. In the current study, we investigated the effect of ALAEm on this pathway in placental tissues and found that changes in the EGFR and VEGFR2 proteins induced the phosphorylation of their downstream pathway, the PI3K-AKT1 pathway, which have been shown to mediate inflammation (15), inhibit the infiltration and implantation of ectopic endometrium by decreasing the expression of Caspase-3 (85), and increase the expression of CCND1 (40). Previous studies on the mechanism of action of additives have shown that additives may improve sow reproduction through different mechanisms of action, e.g., supplementation of sows with *Artemisia annua* enhances inflammatory and innate immune responses by inhibiting TLR4/NF-κB and MAPK pathways, and ultimately attenuates oxidative and inflammatory responses associated with neonatal and early weaning. Our study have shown that flavonoid active ingredients (e.g., liquiritin), which are widely present in plant extracts such as *Glycyrrhiza uralensis* Fisch. and *Astragalus membranaceus* (Fisch.) Bge., can reduce inflammatory responses by inhibiting the activation of the extracellular signaling-associated kinase 1/2 (ERK1/2)/NF- κB pathway and promoting the activation of the Nrf2/Keap1 pathway (86), which are downstream of the PI3K-AKT1 signaling pathway. eNOS, which also belongs to the downstream factors of PI3K-AKT1, promotes the growth of uterine arteries and spiral arteries, which in turn induces VEGF to promote neointima formation (87). Therefore, it is inferred that ALAEm may promot placental growth and angiogenesis through the EGFR/VEGFR2-PI3K-AKT1-eNOS pathway and regulate placental function by regulating responses such as inflammation, apoptosis, and oxidation through EGFR/VEGFR2-PI3K-AKT1 pathway, in which the flavonoids in ALAEm play a key role.

**Figure 10:**
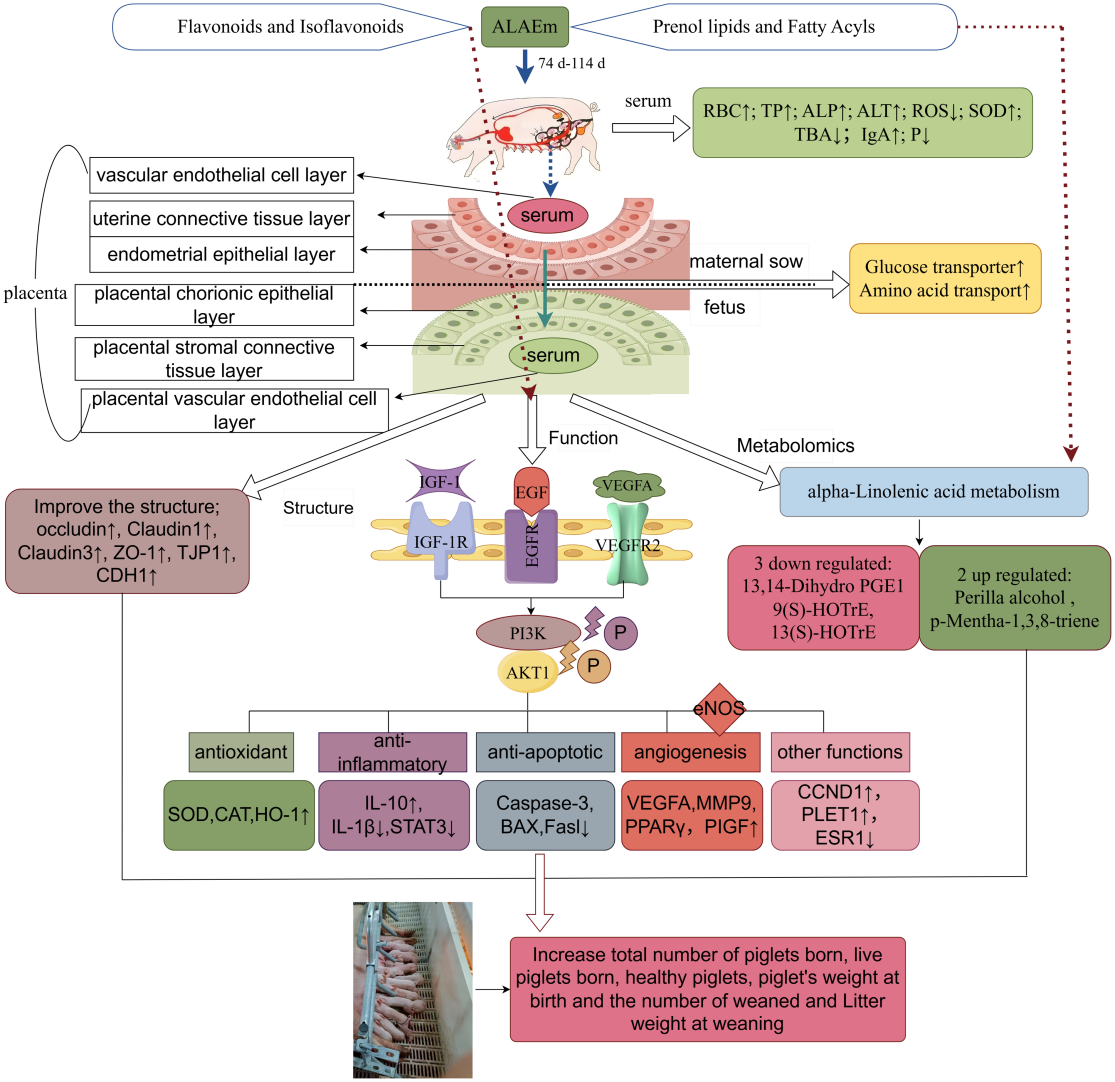
Schematic diagram of study overview and the mechanism of action of ALAEm in improving the reproductive performance of sows (by Figdraw).

Metabolomics analysis identified two upregulated and three downregulated differential metabolites. The downregulated metabolites included the oxidized lipids 13(S)-HOTrE, 9(S)-HOTrE, and 13,14-dihydro PGE1, which are associated with fatty acid metabolism and fatty acid transport into the placenta. These substances can be directly transported into the fetal circulation, where they meet the extensive metabolism of the placenta throughout gestation by oxidatively producing ATP (88, 89), a process that may induce oxidative stress in the placenta (90). Both 13(S)-HOTrE and 9(S)-HOTrE play an important role in the inflammatory response (91). A previous study showed that 13(S)-HOTrE was upregulated in mares with endometritis compared with normal mares, and 13(S)-HOTrE was identified as a potential biomarker for the diagnosis of endometritis in mares (92). Additionally, 9(S)-HOTrE levels were elevated in plasma of postpartum dairy cows with the presence of uterine inflammation (93). 13,14-Dihydro PGE1 is an *in vivo* metabolite of PGE1. PGs are important regulators of pregnancy and are synthesized mainly from arachidonic acid and linoleic acid in the placenta; PGs reduce mitogenic activity induced by corticosteroid administration (94), improve lipid metabolism of the vascular wall (95), inhibit platelet aggregation, and constrict vasoconstriction (96). Metabolism of linoleic acid to PGs in the placenta will help ensures a positive pregnancy outcome (97, 98). p-Mentha-1,3,8-triene and perilla alcohol are upregulated in the ALAEm group and are generally found in essential oils with antimicrobial and antioxidant activities (94, 99), which reduce VEGF release in cancer cells, stimulate endothelial cell expression of angiopoietin 2 (Ang2), and inhibit tumor formation (100). These differential metabolites are mainly involved in the alpha-linolenic acid metabolism pathway; abnormalities in this pathway may lead to problems such as aberrant lipid metabolism in the body and decreased immunity. Severe abnormalities in lipid metabolism can increase the incidence of hypertension and preeclampsia during pregnancy. Some studies have shown that adding lipids to the lactation diet of sows improves milk fat secretion, which is beneficial to the development of lactating piglets and subsequent reproduction of sows (101). Increasing the intake of alpha-linolenic acid (ALA) during lactation increases litter size and pig weaning weights without affecting piglet survival or subsequent sow performance (102, 103). Fatty acids are ligands for the transcription factor PPAR, and reduced PPAR expression is associated with placental developmental disorders (e.g., delivery of animals with a low birth weight). ALA promotes the expression of PPAR*γ*, which regulates angiogenesis in the placenta (104). ALA was also detected in ALAEm components. ALA regulates genes related to oxidative stress and inflammation by affecting NF-κB (105); it inhibits pro-inflammatory factor formation such as TNF-*α* and IL-1β, which is consistent with our findings. The lipid active ingredient glycyrrhizic acid inhibits activation of the NF-κB signaling pathway, modulates immune cells at the maternal-fetal interface, attenuates endometrial pyroptosis and inflammation during pregnancy (106, 107), and significantly inhibits the development of endometriosis (108). Glabridin is involved in the regulation of genes related to lipid metabolism and has significant PPAR-γ binding activity (109). Therefore, we hypothesize that ALAEm-mediated alteration of metabolites related to the alpha-linolenic acid metabolism pathway in sow placental tissues may regulate sow reproduction. More experiments are needed to verify this possibility. A diagram summarizing the workflow and findings of this study is provided in Figure 10.

## 5 Conclusion

In this study, we showed that dietary addition of ALAEm in late gestation sows improved the levels of factors in the serum and cord blood of sows and improved placental structure. We inferred that ALAEm may attenuate the levels of inflammation, oxidation and apoptosis in placental tissues and promote placental growth, angiogenesis, and nutrient transport through the EGFR/VEGFR2-PI3K-AKT1 pathway. Additionally, lipid components of ALAEm may alter the levels of placental lipid metabolites by lipids through alpha-linolenic acid metabolism.

This study has several limitations. First, we did not conduct experimental examination of the potential regulatory effects of flavonoids or lipid-based active components on sow reproductive performance. Second, because there is no uniformly used positive drug used to regulate sow reproduction in late gestation in modern swine farms and because many factors affect sow reproduction, we were unable to include positive and model control groups. Third, further experiments are needed to investigate the effects of ALAEm on piglet growth performance, nutrients in umbilical cord blood, and the number of days to estrus after weaning of sows to more convincingly justify the results of this article exploring the mechanism of ALAEm in regulating sow postpartum performance and piglet growth. In future studies we will explore the role of ALAEm in combination with other additives and further investigate the mechanism of action of ALAEm through the use of inhibitors or gene silencing.

## Data Availability

The original contributions presented in the study are included in the article/Supplementary material, further inquiries can be directed to the corresponding author.

## Footnotes

^1^ http://chemrich.fiehnlab.ucdavis.edu/

## Supplementary material

Supplementary File 1 A total of 803 active components in ALAEm.

Supplementary File 2 The top 30 components of ALAEm-secondary mass spectrometry.

Supplementary File 3 1532 metabolites.

Supplementary File 4 The box line diagrams of five differential metabolites.

## Glossary

ALAEm: A natural plant complex consisting of *Angelica sinensis* (Oliv.) Diels, *Glycyrrhiza uralensis* Fisch., *Astragalus membranaceus* (Fisch.) Bge., *Eucommia ulmoides* Oliv., *Atractylodes macrocephala* Koidz., *Populus tomentosa* Carr., *Perilla frutescens* (L.) Britt., Gynostemma pentaphyllum and *Leonurus japonicus* Houtt.
VEGFA: Vascular endothelial growth factor A
MMP9: Matrix metalloproteinase 9
PPARγ: Peroxisome proliferator-activated receptor gamma
PIGF: Placental growth factor
VEGFR1: Vascular endothelial growth factor receptor 1
VEGFR2: Vascular endothelial growth factor receptor 2
EGF: Epidermal growth factor
IGF-1: Insulin-like growth factor 1
EGFR: Epidermal growth factor receptor
IGF1R: Insulin-like growth factor 1 receptor
TGFβR1: Transforming growth factor beta receptor 1
FGFR: Fibroblast growth factor receptor
CDH1: Cadherin 1
CTNNB1: Catenin beta 1
GLUT1: Glucose transporter 1
SNAT1: Sodium-coupled neutral amino acid transporter 1
SNAT2: Sodium-coupled neutral amino acid transporter 2
PLET1: Placenta-expressed transcript 1
ESR1: Estrogen receptor 1
CCND1: Cyclin D1
eNOS: Endothelial nitric oxide synthase
UPLC-MS/MS: Ultra-performance liquid chromatography coupled with tandem mass spectrometry
FC: Fold change
VIP: Variable importance on projection
IUGR: Intrauterine growth retardation
PGE1: Prostaglandin E1
ALA: Alpha-linolenic acid
