# Supplementary figures and images for: Biochemical impact of ALAEm supplementation in late gestation on the reproductive performance of sows
Source: Front Vet Sci. 2025 Apr 23;12:1548263. doi: 10.3389/fvets.2025.1548263 (PMC12055862; doi:10.3389/fvets.2025.1548263)

Supplementary Material 2-The top 30 components of ALAEm-secondary mass spectrometry


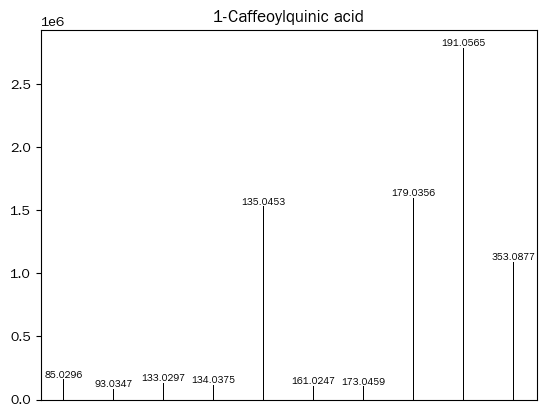

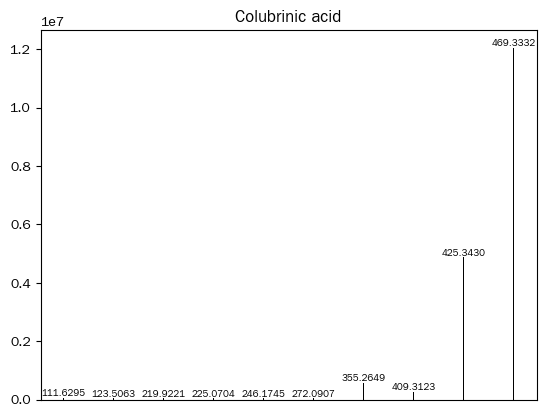

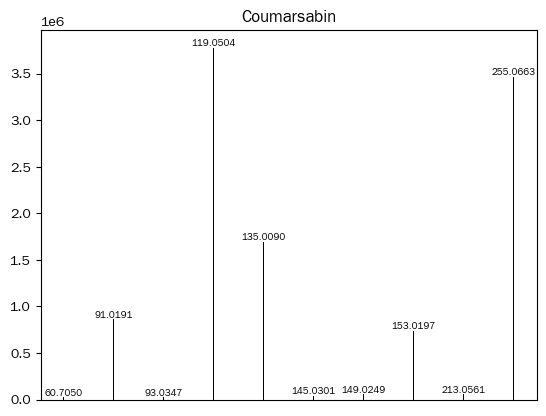

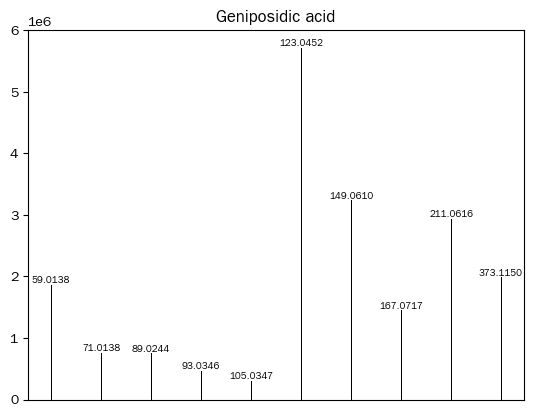

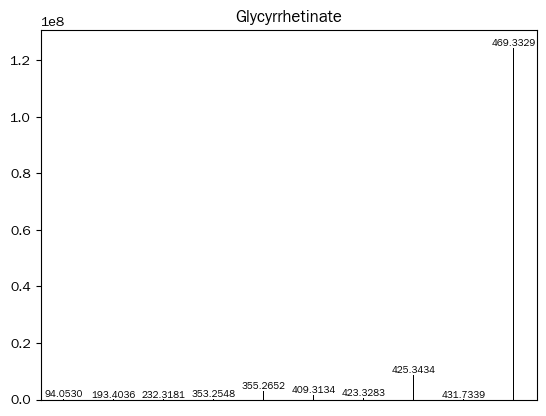

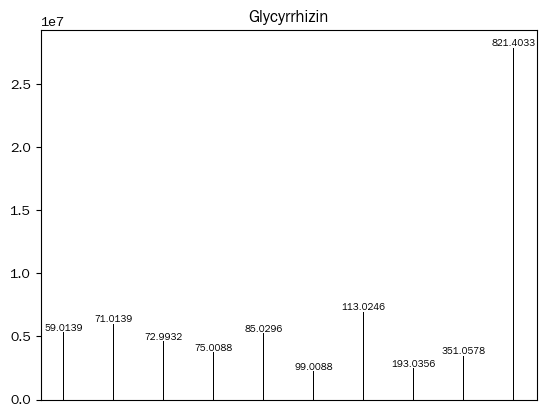

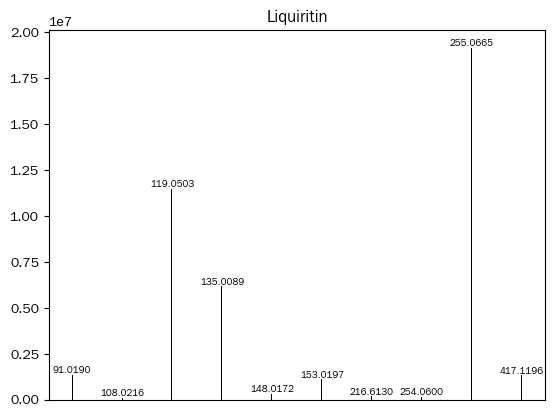

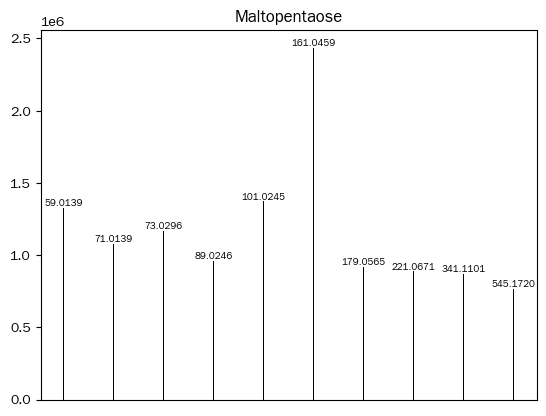

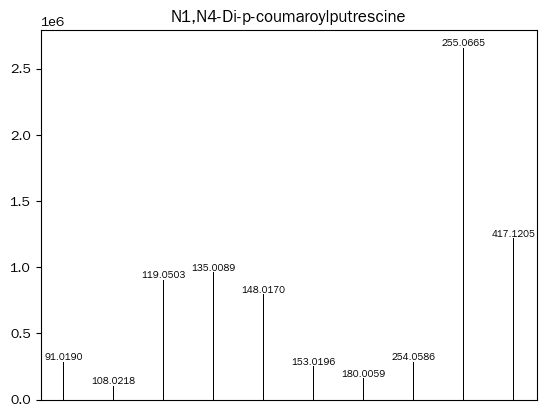

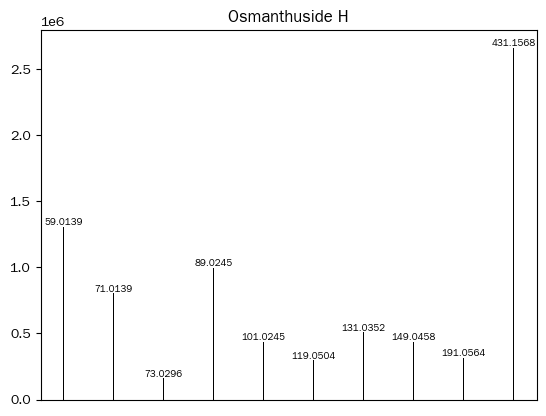

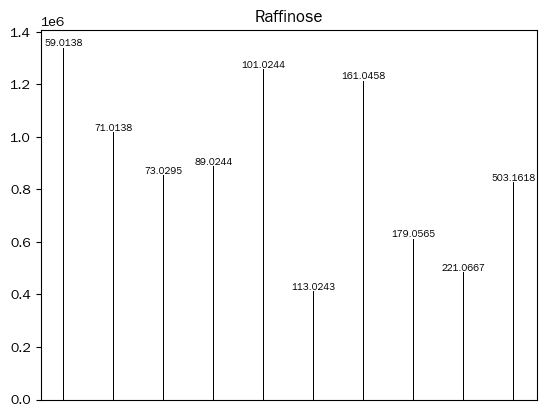

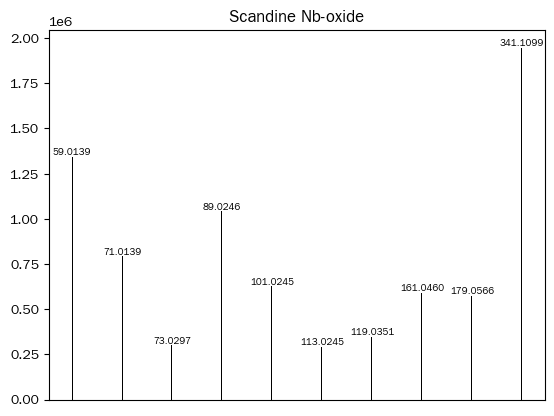

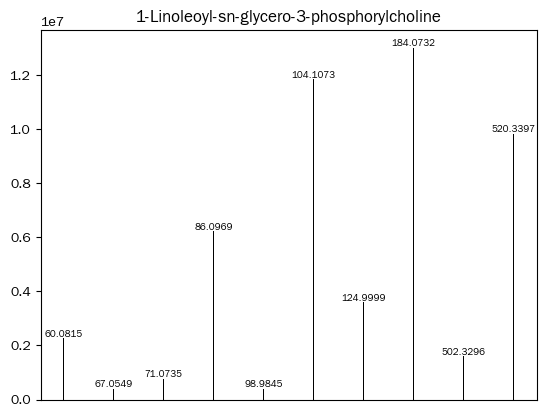

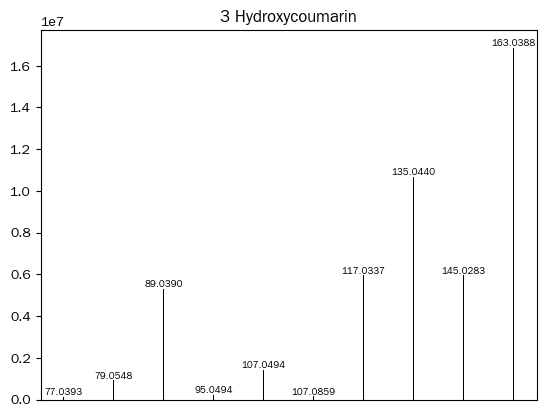

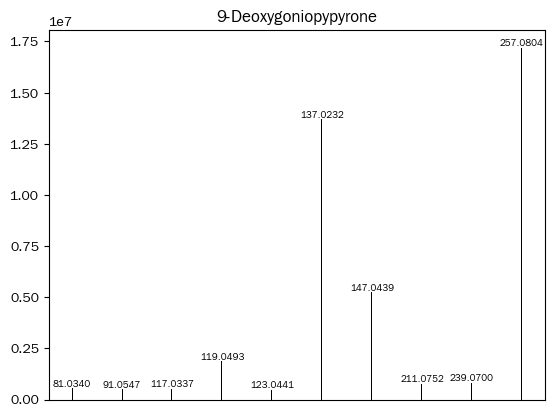

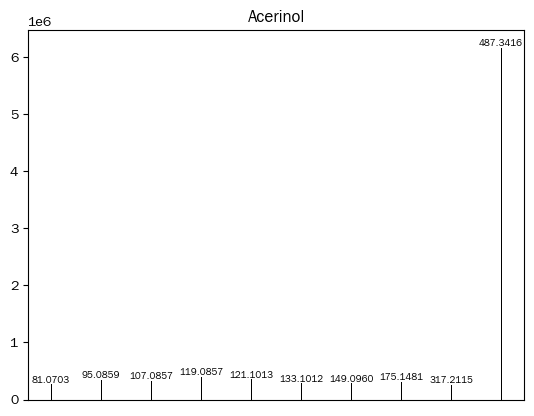

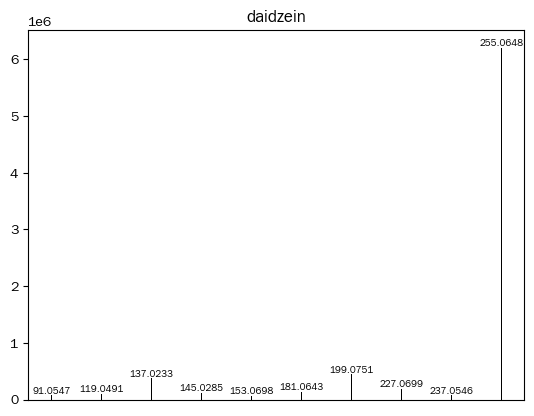

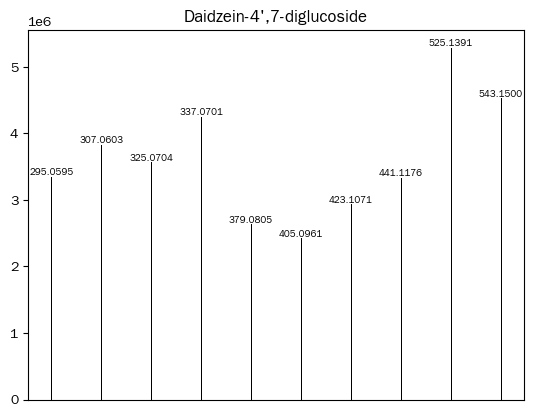

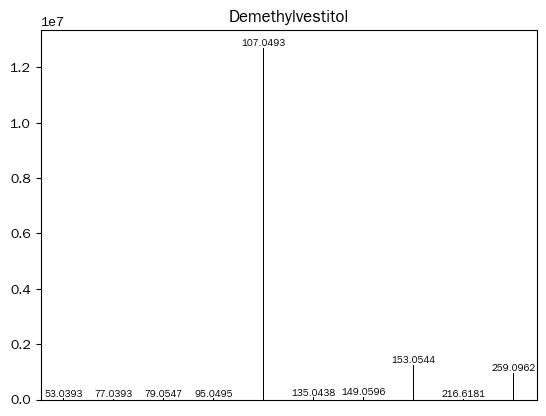

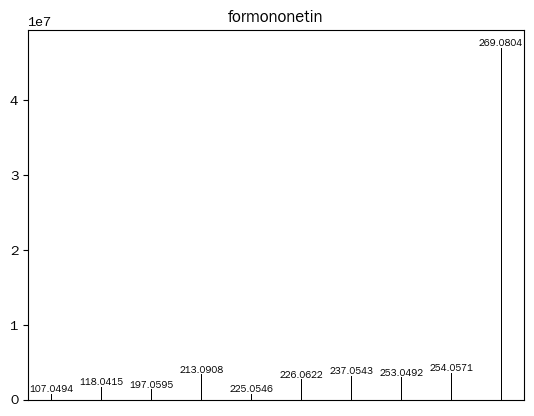

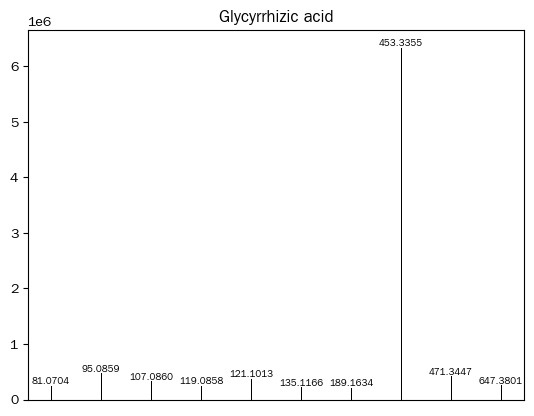

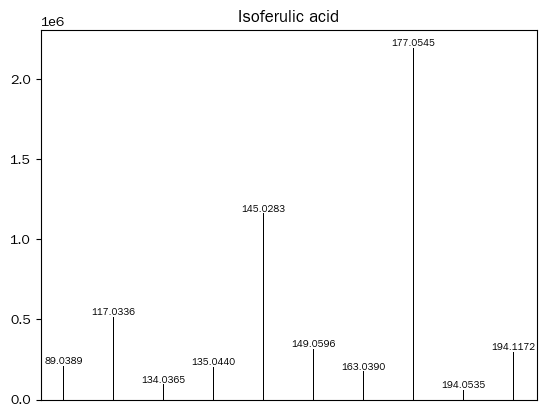

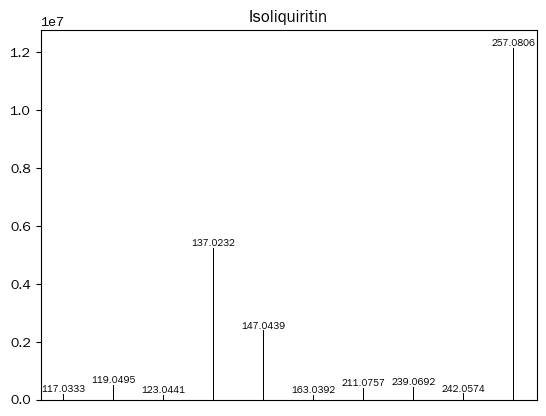

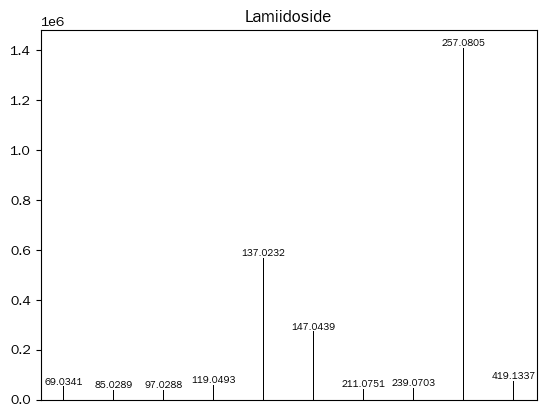

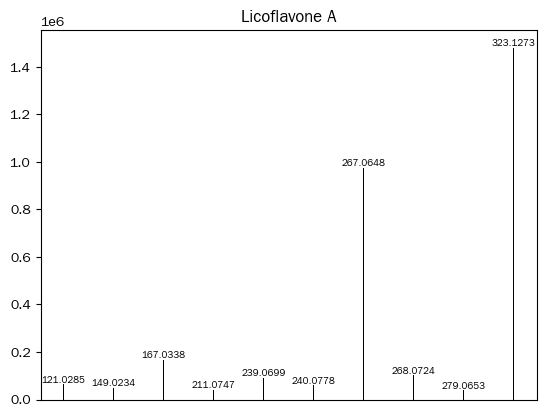

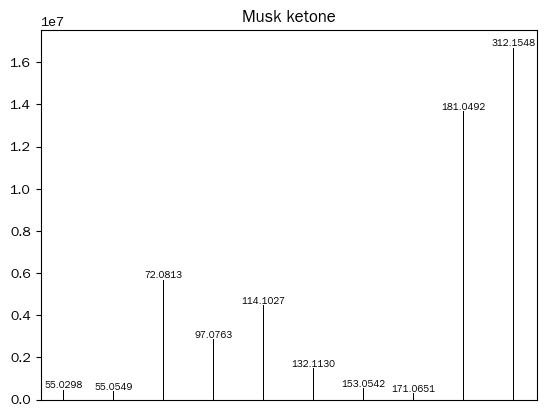

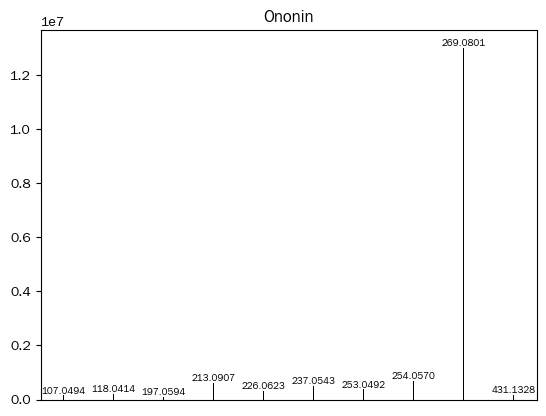

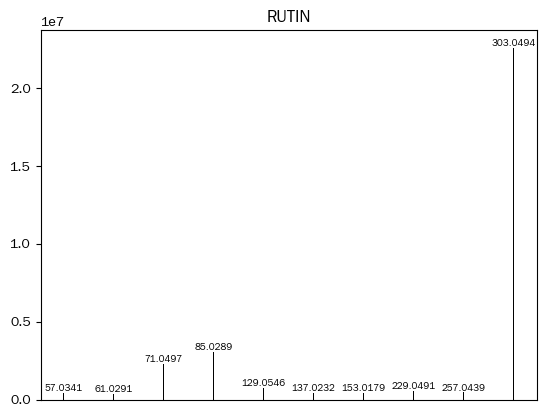

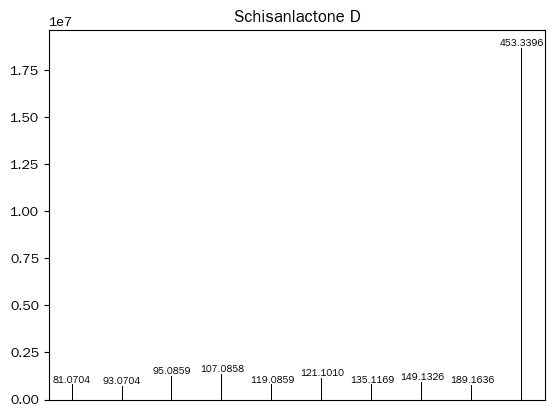


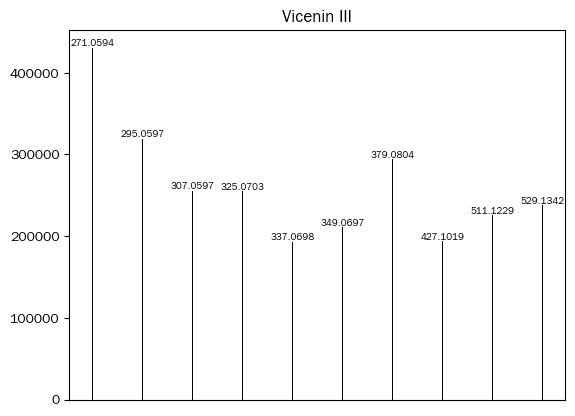

Supplement: Supplementary File 2 — The top 30 components of ALAEm-secondary mass spectrometry. [file Supplementary_file_2.docx]

Supplementary Material 4-The box line diagrams of five differential metabolites:


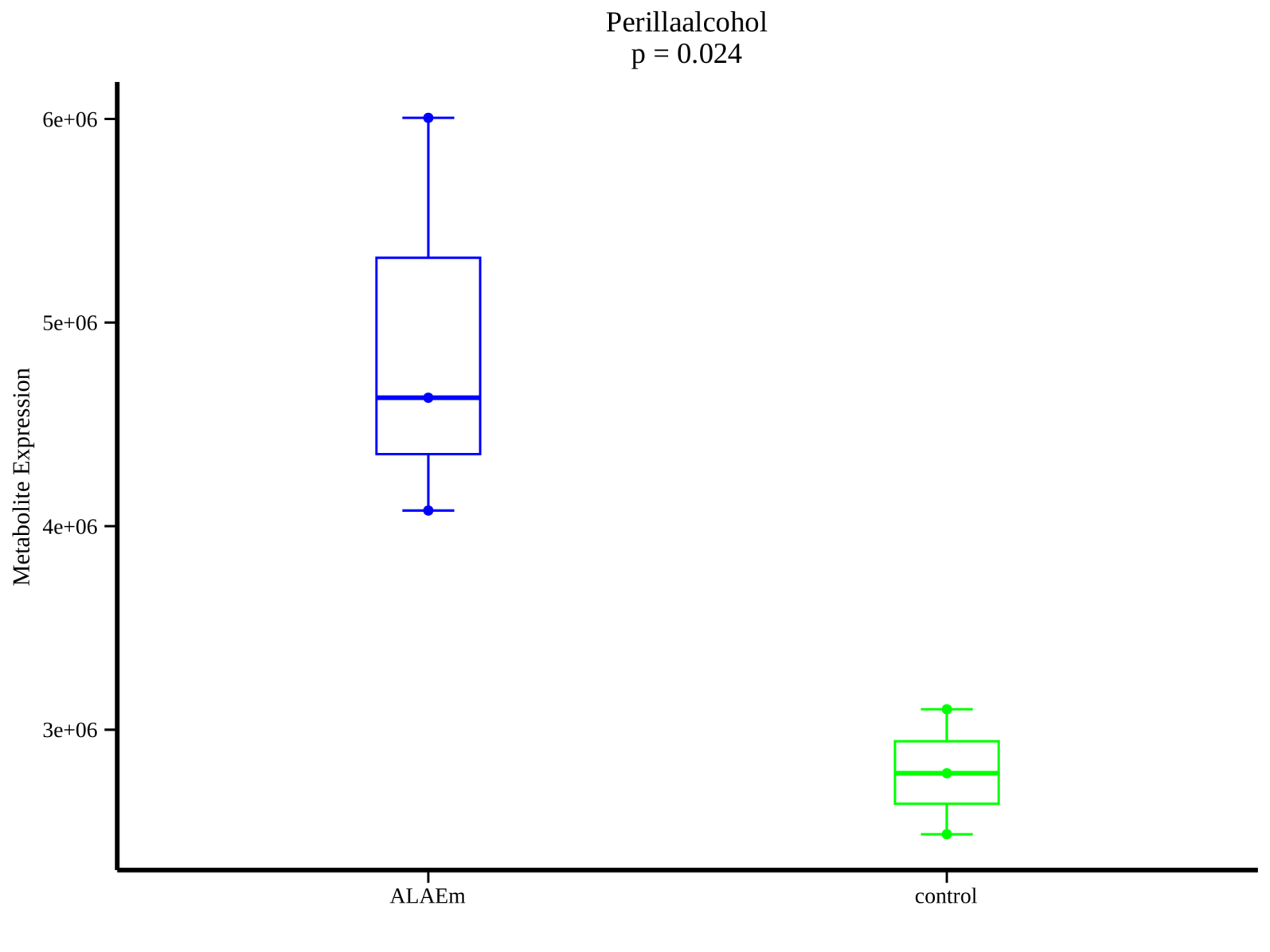

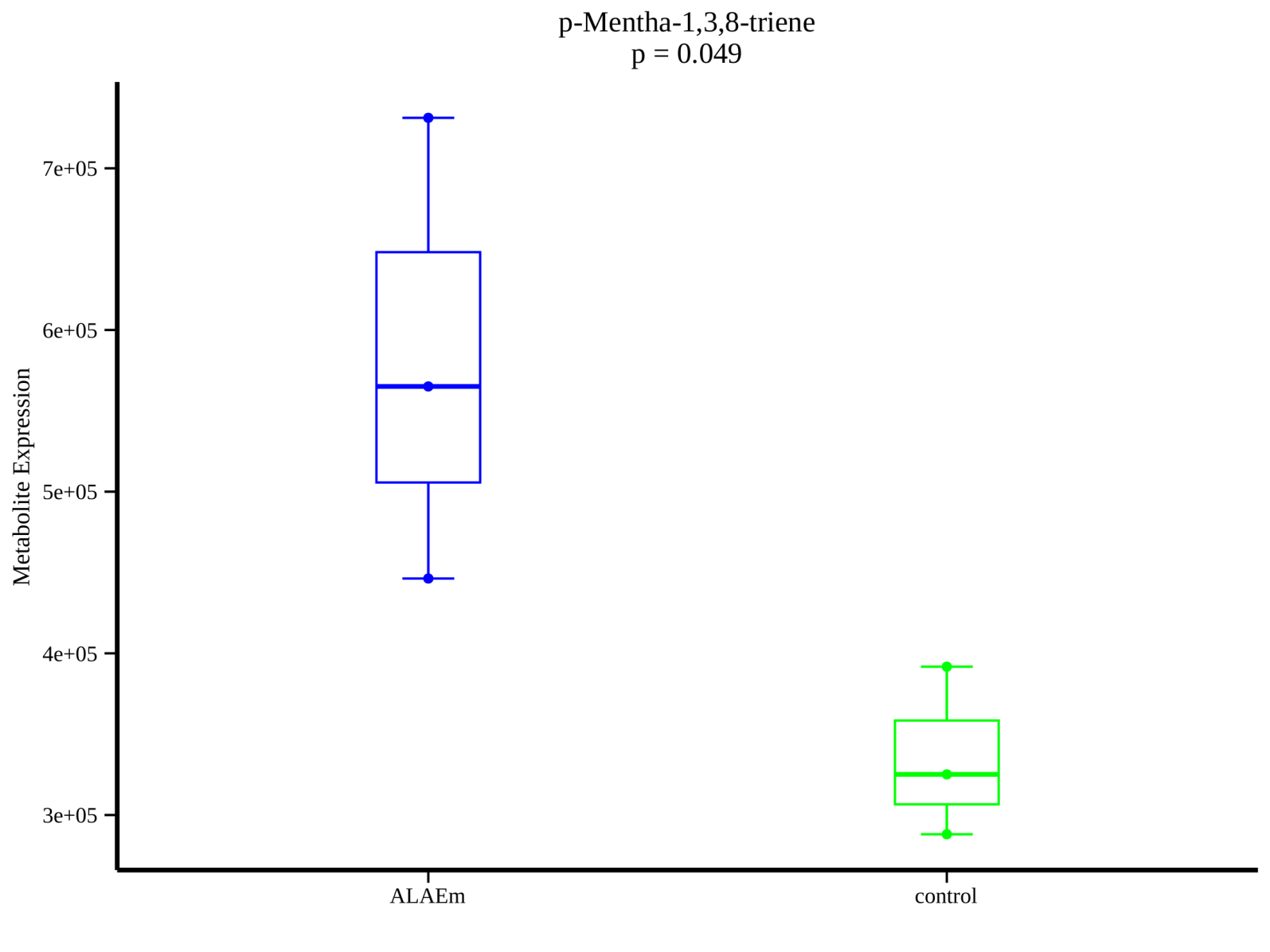

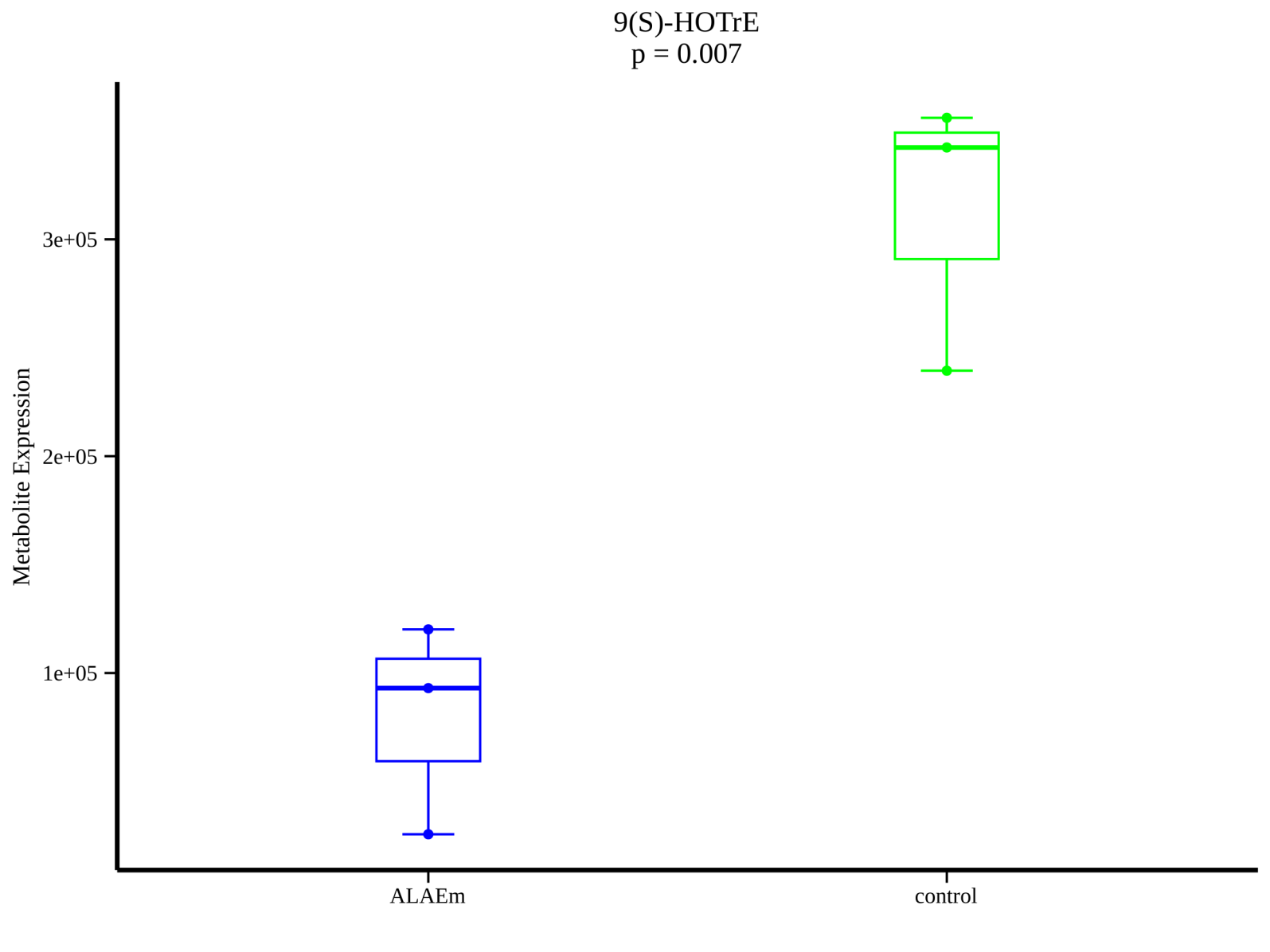

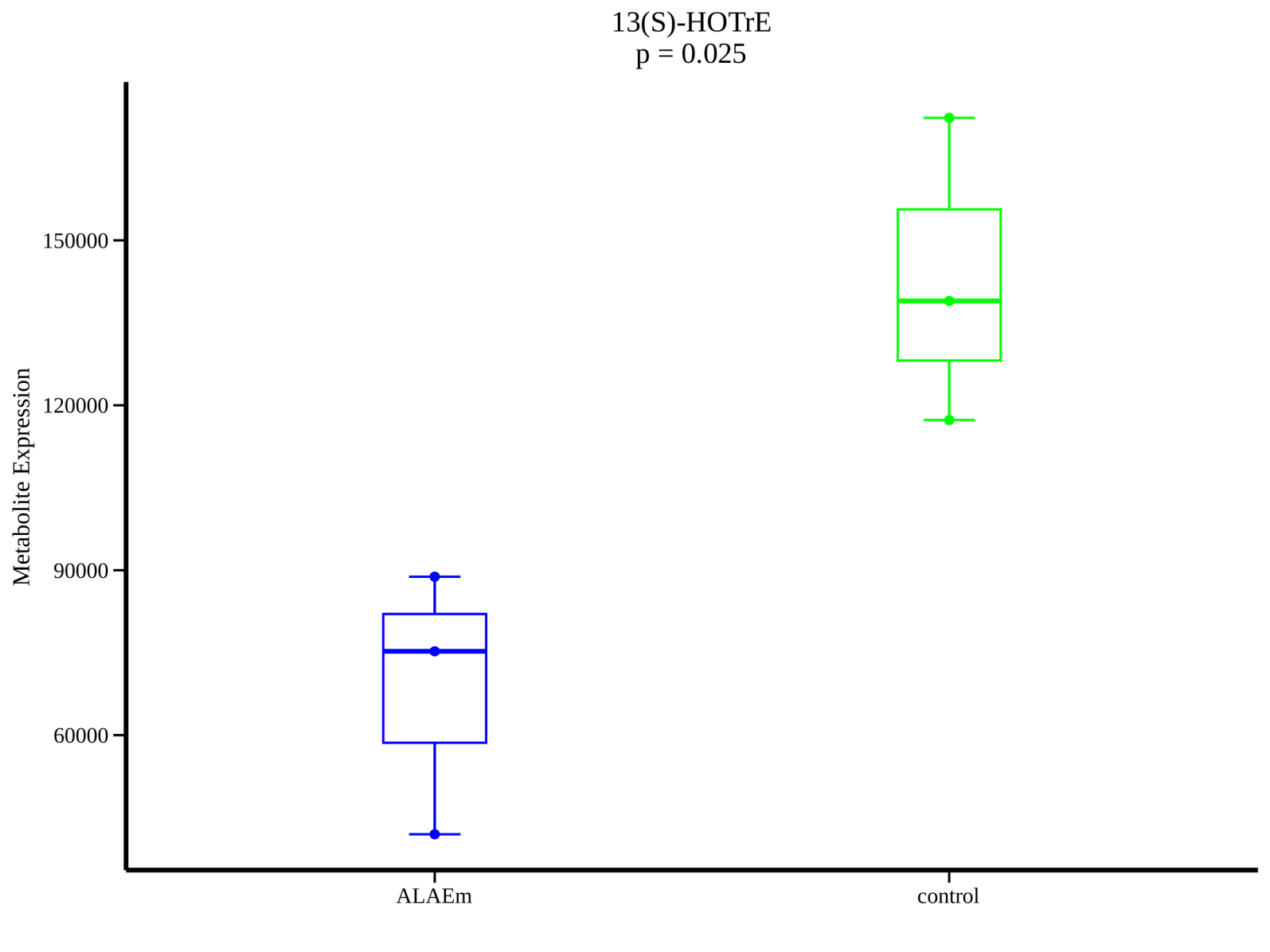

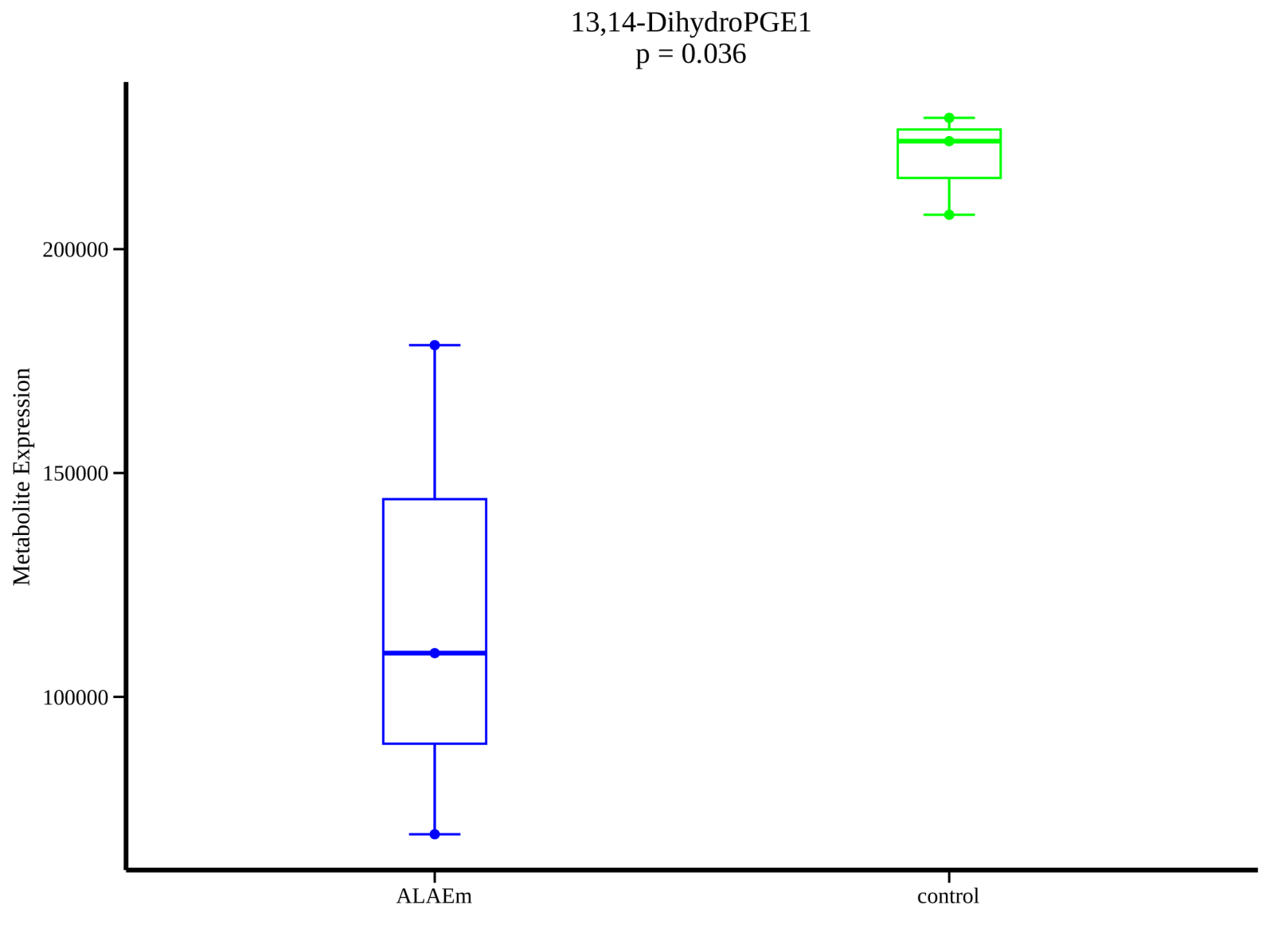

Supplement: Supplementary File 4 — The box line diagrams of five differential metabolites. [file Supplementary_file_4.docx]
